# Cysteine facilitates the lignocellulolytic response of *Trichoderma* *guizhouense* NJAU4742 by indirectly up-regulating membrane sugar transporters

**DOI:** 10.1186/s13068-023-02418-9

**Published:** 2023-10-27

**Authors:** Yang Liu, Tuo Li, Han Zhu, Yihao Zhou, Qirong Shen, Dongyang Liu

**Affiliations:** 1 Jiangsu Provincial Key Lab of Solid Organic Waste Utilization, Jiangsu Collaborative Innovation Center of Solid Organic Wastes, Educational Ministry Engineering Center of Resource-Saving Fertilizers, Nanjing, People’s Republic of China; 2 College of Resources and Environmental Sciences, Nanjing Agricultural University, Nanjing, 210095 Jiangsu People’s Republic of China

**Keywords:** *T.guizhouense* NJAU4742, Sugar transporter, Lignocellulolytic response, Multiomics, Protein-DNA interactions, Gene manipulation, Glutathionylation

## Abstract

**Background:**

Filamentous fungi possess a rich CAZymes system, which is widely studied and applied in the bio-conversion of plant biomass to alcohol chemicals. Carbon source acquisition is the fundamental driver for CAZymes-producing sustainability and secondary metabolism, therefore, a deeper insight into the regulatory network of sugar transport in filamentous fungi has become urgent.

**Results:**

This study reports an important linkage of sulfur assimilation to lignocellulose response of filamentous fungus. Inorganic sulfur addition facilitated biodegradation of rice straw by *Trichoderma* *guizhouense* NJAU4742. Cysteine and glutathione were revealed as major intracellular metabolites responsive to sulfur addition by metabolomics, cysteine content was increased in this process and glutathione increased correspondingly. Two membrane sugar transporter genes, *Tgmst*1 and *Tgmst*2, were identified as the critical response genes significantly up-regulated when intracellular cysteine increased. *Tgmst*1 and *Tgmst*2 were both positively regulated by the glucose regulation-related protein (GRP), up-regulation of both *Tgmst*1 and *Tggrp* can cause a significant increase in intracellular glucose. The transcriptional regulatory function of GRP mainly relied on GSH-induced glutathionylation, and the transcription activating efficiency was positively related to the glutathionylation level, furthermore, DTT-induced deglutathionylation resulted in the down-regulation of downstream genes.

**Conclusions:**

Inorganic sulfur addition induces a rise in intracellular Cys content, and the conversion of cysteine to glutathione caused the increase of glutathionylation level of GRP, which in turn up-regulated *Tgmst*1 and *Tgmst*2. Subsequently, the sugar transport efficiency of single cells was improved, which facilitated the maintenance of vigorous CAZymes metabolism and the straw-to-biomass conversion.

**Supplementary Information:**

The online version contains supplementary material available at 10.1186/s13068-023-02418-9.

## Background

The bio-production of biofuels and chemicals from plant biomass is one of the most promising ways to solve the current energy and environmental crisis. Filamentous fungi commonly own abundant lignocellulases systems and complex regulatory networks, which can regulate the lignocellulolytic carbohydrate active enzymes (CAZymes) expression flexibly according to different natural carbon sources [1]. Meanwhile, filamentous fungi play an irreplaceable role in terrestrial ecosystems' carbon cycle and soil microbial ecology's stability by degrading the plant residues [2]. Plant biomass components are mainly composed of cellulose (40–50%), hemicellulose (20–40%), and lignin (20–30%) [3]. Filamentous fungi display a considerable utilization capacity of pentose (C5) and hexose (C6), resulting in the efficient degradation of the complete components of plant biomass. Therefore, they are widely used for agricultural solid waste management, especially for the typical representatives: *Trichoderma* *sp.*, *Aspergillus* *sp.*, and *Penicillium* *sp.* [4, 5].

The degradation efficiency of filamentous fungi on lignocellulosic materials depends on synergistic work of cellulase, hemicellulase, and auxiliary activity (AA) family proteins [6, 7]. Cellulases are composed of endoglucanases (EG), cellobiohydrolase (CBH), and β-glucosidase (BG), which hydrolyze cellulose to form cellobiose and glucose. In contrast, hemicellulases can hydrolyze polymers of xylose, arabinose, or mannose, such as xylan and glucomannan [8]. Therefore, the activity of these enzymes is an important parameter to evaluate biodegradation efficiency. CAZymes expression level is regulated by elaborate regulatory networks, which mainly contain transcription factor XLNR/ACE2, transcriptional repressor ACE1, CCAAT binding complexes HAP2/3/5, and glucose repression protein CRE1 and AREA [9]. In *Trichoderma* *sp.* and *Aspergillus* *sp.*, XLNR/XYR1/CLR1/CLR2 are slated to regulate the expression of cellulase and hemicellulase genes [10].

The traditional conversion of biomass into biofuels requires saccharification in early stage and crude sugars fermented by *Saccharomyces* *cerevisiae* for alcohol production. Recently, efficient alcohol fermentation by filamentous fungi was successful through metabolic engineering, and the bio-conversion of plant biomass into alcohol chemicals can be achieved in one step [11]. Therefore, exploring a method to improve the filamentous fungi's sugar transport capacity will provide support for the program. The intracellular transport efficiency of lignocellulosic hydrolysates (mainly glucose, xylose, and cellobiose) is the primary driving force for enzyme production sustainability and secondary metabolism. The GPCR/cAMP/PKA signaling cascade is responsible for recognizing extracellular sugar and activating sugar transporters in fungi [12]. Git3/Gpr/HXT are identified as the main glucose sensor, which can sense extracellular glucose and activate adenylate cyclase (AC) through G-protein [13, 14]. AC can convert ATP into cyclic adenosine monophosphate (cAMP), and protein kinase A (PKA) is the cAMP receptor, which initiates sugar transporters through phosphorylation [15], and this process involves protein–protein interactions. Sugar transporter in fungi mainly includes glucose transporter GLT1, xylose transporter XYT1, arabinose transporter LAT1, and xylose and arabinose transporter XAT1 [16], and all of them are homologous to the major facilitator superfamily proteins (MFS) [17, 18].

*Trichoderma* *guizhouense* NJAU4742 is a beneficial fungus isolated from compost, which can secrete various plant-promoting factors such as SWO and IAA and possesses a rich CAZymes system, so it has a high industrial and agricultural value [19–22]. Here, we report the promotional effects of inorganic sulfides on lignocellulolytic response of NJAU4742, which included an increase in biomass and multiple enzyme activities. Through genetic manipulation, transcriptomic analyses, and protein-DNA interaction studies, we uncovered that sulfur assimilation products inducing the upregulation of sugar transporters were direct contributors and revealed the specific regulation modes. This study provides insights into the carbon source metabolism regulatory network of filamentous fungi and supports for metabolic engineering.

## Results

### Inorganic sulfide significantly enhanced the lignocellulose utilization ability of NJAU4742

**Fig. 1:**
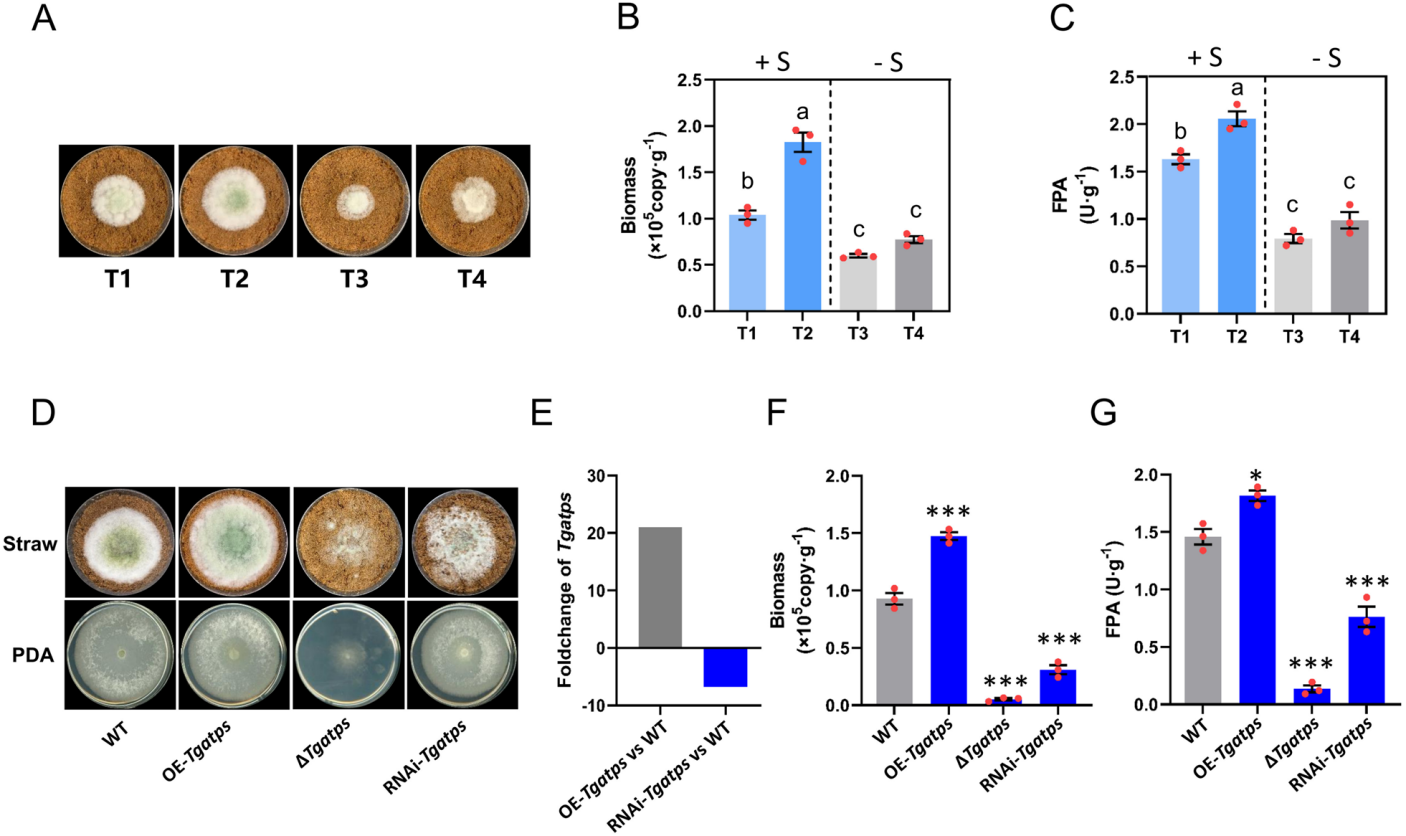
Promotional effect of inorganic sulfide on the lignocellulolytic response of NJAU4742. **A** Inorganic sulfide content was only variable for the four treatments: T1: (NH_4_)_2_SO_4_ + 2 KH_2_PO_4_, T2: 2 × T1; T3: (NH_4_)_2_HPO_4_ + K_2_HPO_4_, T4: 2 × T3 (see Methods for details). WT of NJAU4742 grown on the straw with different sulfate content at 28 °C for 3 days. **B** The copies of the NJAU4742 genome were determined by absolute qPCR to characterize hyphae biomass in different treatments. The left Y axis was raw Ct, and the right Y axis was genome copies (biomass). **C** FPA in different treatments, the strain was grown at 28 °C for 5 days. **D** Growth status of WT, OE-*Tgatps*, ∆*Tgatps*, and RNAi-*Tgatps* on MM + straw and PDA, strains were grown at 28 °C 4 days. **E** FoldChange of OE-*Tgatps* and RNAi *Tgatps* relative to WT, 21.04 folds up-regulation of *Tgatps* in OE-*Tgatps*, 6.76 folds down-regulation in RNAi-*Tgatps*. **F** Hyphae biomass of WT, OE-*Tgatps*, ∆*Tgatps* *and* RNAi-*Tgatps* on straw medium at 28 °C for 5 days. **G** FPA of WT, OE-*Tgatps*, ∆*Tgatps*, and RNAi-*Tgatps* on MM + straw, strains were grown at 28 °C for 5 days. Bars represent mean ± SEM, with n = 3 biological repeats; red dots resemble values from individual experiments. ANOVA was conducted in (**B**, **C**), Tukey's HSD test was used for post hoc comparisons, and the letters “a”, “b”, and “c” were used for significance exhibition. Inorganic sulfide has a significant effect on biomass and FPA (*P* < 0.05). Student’s *t*-testing was conducted in (**F**, **G**), ***significant difference to WT at two-tailed *P* = 0.00027 (**F**, OE-*Tgatps*), 0.00001 (**F**, ∆*Tgatps*), 0.00011 (**F**, RNAi-*Tgatps*); *significant difference to WT at two-tailed *P* = 0.015 (**G**, OE-*Tgatps*), ***significant difference to WT at two-tailed *P* = 0.00001 (**G**, ∆*Tgatps*), 0.00022 (**G**, RNAi-*Tgatps*)

Four adjusted mineral mediums (MM) were set up to evaluate the effect of inorganic sulfur on the lignocellulose utilization ability of NJAU4742: sulfur addition treatment (T1 and T2), T1: (NH_4_)_2_SO_4_ (1 mmol) + KH_2_PO_4_ (2 mmol), T2: 2 × T1; No sulfur addition treatment (T3 and T4), T3: (NH_4_)_2_HPO_4_ (1 mmol) + K_2_HPO_4_ (1 mmol), T4: 2 × T3, other mineral nutrients and pH were adjusted at a same level, various forms of sulfides and soluble sugar in straw have been removed as much as possible. The growth status of NJAU4742 exhibited variation in different treatments (Fig. 1A). The results indicated that the biomass in different treatments increased with content of sulfur, and the highest value (1.83 × 10^5^ copy g^−1^) was obtained in T2, and the lowest biomass (0.60 × 10^5^ copy g^−1^) was found in T3 (Fig. 1B). Filter paper enzyme activity (FPA) can be used to characterize overall CAZymes activity. The FPA of T1 (1.67 U g^−1^) and T2 (2.05 U g^−1^) were significantly greater than that of T3 (0.79 U g^−1^) and T4 (0.99 U g^−1^) (Fig. 1C), and similar trends were obtained in EG, CBH, and Xylanase activity (Additional file 1: Fig. S5A). The results showed that the enzyme activity and biomass of sulfur treatment (T1 and T2) were generally higher than those of no sulfur addition treatment (T3 and T4), indicating that sulfur may have a potential promoting effect on lignocellulosic response of NJAU4742. The difference between T3 and T4 was not significant, indicating that increasing mineral nutrients without sulfur cannot significantly promote lignocellulose utilization.

To verify this hypothesis, the key sulfur assimilating enzyme ATP sulfatase gene (*Tgatps*, KEGG ID: K00958, GENE ID: A1A109512.1, NCBI ID: OPB38572.1) was overexpressed in OE-*Tgatps* (21.04 folds up-regulated, Fig. 1E) and deleted in ∆*Tgatps*. Concurrently, *Tgatps* was silenced by constructing the pSilent-*Tgatps* vector to express RNA-induced silencing complex (RISC) (Fig. 1D; Additional file 1: Fig. S2A). The biomass of OE-*Tgatps* was significantly higher than that of WT, and deletion or silence of *Tgatps* caused a sharp reduction in biomass (Fig. 1F). The CAZymes of OE-*Tgatps* were significantly higher than that of WT, while it declined rapidly both in ∆*Tgatps* and RNAi-*Tgatps* relative to WT (Fig. 1G; Additional file 1: Fig. S5B). Besides, the growth of RNAi-*Tgatps* in PDA medium was not affected but worse in MM + straw, which indicated that the reduction of sulfur assimilation would affect the straw utilization capacity of NJAU4742 and did not affect the basic cellular metabolism in full-nutrient medium.

### The increase of intracellular Cys and Met facilitated the lignocellulolytic response of NJAU4742

**Fig. 2:**
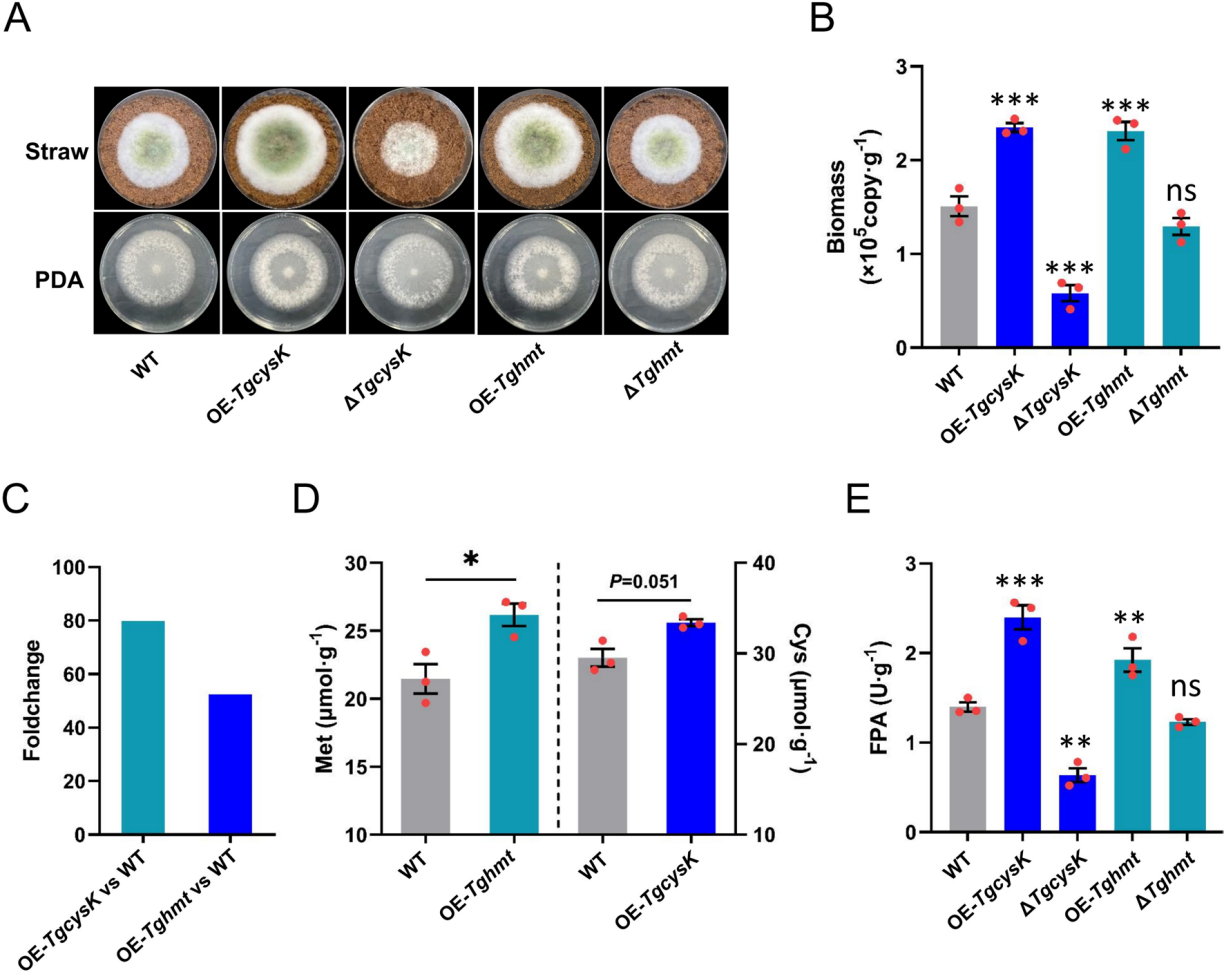
The effect of increasing intracellular Cys and Met content on biomass and CAZymes activities of NJAU4742. **A** The growth status of WT, OE-*TgcysK*, ∆*TgcysK*, OE-*Tghmt*, and ∆*Tghmt* on MM + straw and PDA, grown at 28 °C for 3 days, note that growth of strains on PDA was not different, but there was different on MM + straw. **B** Hyphae biomass of WT, OE-*TgcysK*, ∆*TgcysK*, OE-*Tghmt* and ∆*Tghmt*, strains were grown on MM + straw for at 28 °C 4 days. **C** FoldChange of *TgcysK* (52.35 folds up-regulation) in OE-*TgcysK* relative to WT and FoldChange of *Tghmt* (79.75 folds up-regulation) in OE-*Tghmt* relative to WT. **D** Intracellular Cys content of OE-*TgcysK* and intracellular Met content of OE-*Tghmt*, strains were grown on PDA at 28 °C 3 days. **E** FPA of WT, OE-*TgcysK*, ∆*TgcysK*, OE-*Tghmt*, and ∆*Tghmt*. Bars represent mean ± SEM, with n = 3 biological repeats; red dots resemble values from individual experiments. Student’s *t*-testing was conducted in (**B**, **D**, **E**), ***significant difference to WT at two-tailed *P* = 0.00011 (**B**, OE-*TgcysK*), 0.00017 (**B**, ∆*TgcysK*), 0.00047 (**B**, OE-*Tghmt*), ns = no statistical difference to WT at two-tailed *P* = 0.23 (**B**, ∆*Tghmt*); *significant difference to WT at two-tailed *P* = 0.020 (**D**, OE-*Tghmt*), no statistical difference to WT at two-tailed *P* = 0.051 (**D**, OE-*TgcysK*); ***significant difference to WT at two-tailed *P* = 0.00081 (**E**, OE-*TgcysK*), **significant difference to WT at two-tailed *P* = 0.0016 (**E**, ∆*TgcysK*), 0.0021 (**E**, OE-*Tghmt*), no statistical difference to WT at two-tailed *P* = 0.73 (**E**, ∆*Tghmt*)

Since inorganic sulfur needs to be assimilated into Cysteine (Cys) before it performs its biological function, and Cys is readily converted to Methionine (Met) [23]. Metabolomic analysis (T1 and T3) showed that Cys and Met metabolism was one of the major metabolism that responded to increased extracellular inorganic sulfide, and the Cys and Met content in T1 were significantly higher than that in T3 (Additional file 1: Fig. S7, Additional file 7: Dataset 3: line190 and line339). The intracellular Cys content in different treatments (T1, T2, T3, and T4) was detected, and results showed that exogenous addition of inorganic sulfide could significantly increase intracellular Cys (Additional file 1: Fig. S1A). The cysteine synthetase gene (*TgcysK*, KEGG ID: K17069, GENE ID: A1A108125.1, NCBI ID: PTB50504.1) and homocysteine methyltransferase (*Tghmt*, KEGG ID: K00547, GENE ID: A1A102309.1, NCBI ID: KKP02592.1) gene were the key enzyme gene for Cys and Met synthesis, respectively. *TgcysK* and *Tghmt* were deleted respectively to obtain the mutants ∆*TgcysK* and ∆*Tghmt*, and both exhibited a normal growth on PDA compared to WT (Fig. 2A), which indicated that sulfur assimilation genes (*TgcysK* and *Tghmt*) were not indispensable for basic metabolism when nutrients sufficient. Considering that exogenously added Cys and Met could be used as preferred carbon and thus induced carbon catabolite repression (CCR), the intracellular Cys and Met were respectively increased by overexpressing key synthase genes. *TgcysK* was overexpressed in OE-*TgcysK* (52.35 folds up-regulated), and *Tghmt* was up-regulated by 79.75 folds in OE-*Tghmt* by comparing with WT (Fig. 2C). The intracellular Cys content in OE-*TgcysK* (26.18 µmol g^−1^) and intracellular Met content in OE-*Tghmt* (33.41 µmol g^−1^) were higher than that in WT (21.48 µmol g^−1^ and 29.54 µmol g^−1^), respectively (Fig. 2D). The biomass and CAZymes of OE-*TgcysK* and OE-*Tghmt* were all significantly higher than those of WT, while these parameters were lower in ∆*TgcysK* than in WT and not significantly different in ∆*Tghmt* and WT. (Fig. 2B, E; Additional file 1: Fig. S5C). These results illustrated that the effect of sulfur assimilation restriction on NJAU4742 growth on straw was not due to a global effect on basic metabolism, and increase of intracellular Cys and Met facilitated the lignocellulolytic response of NJAU4742.

### *Tgmst*1 and *Tgmst*2 were the key response genes for increased intracellular Cys

To reveal how Cys and Met affected lignocellulolytic response of NJAU4742, the total RNA of OE-*TgcysK*, ∆*TgcysK*, OE-*Tghmt*, ∆*Tghmt*, and WT were extracted and transcriptomic analysis was conducted to explore the response genes affected by increased intracellular Cys and Met. After reverse transcription and library construction, low-quality raw reads generated in NGS were filtered out. Clean reads were aligned to the genome database of NJAU4742 and the transcripts number in each sample was obtained. Differential transcripts were screened with *P*-value < 0.05 and FoldChange > 2, and GO and KEGG enrichment analyses of differential transcripts were also performed.

**Fig. 3:**
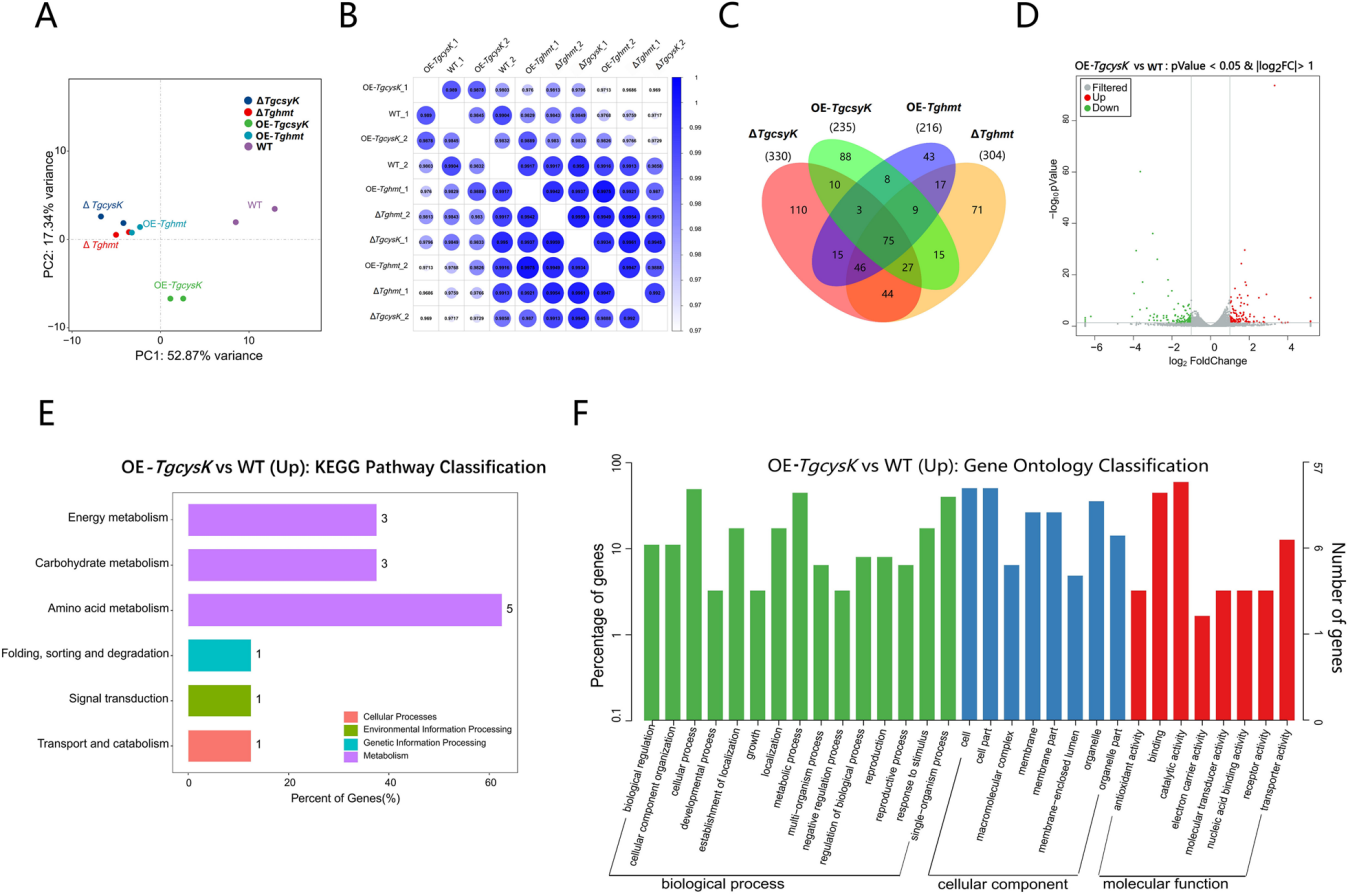
Transcriptome analysis of different strains including WT, OE-*TgcysK*, ∆*TgcysK*, OE-*Tghmt*, and ∆*Tghmt*. **A** PCA of transcriptomes (indicated by dots) in WT, OE-*TgcysK*, ∆*TgcysK*, OE-*Tghmt*, and ∆*Tghmt*. Hyphae were grown at 28 °C for 3 days on MM + straw. Clustering indicated similarity amongst data sets. The number of experiments: 2 (WT, OE-*TgcysK*, ∆*TgcysK*, OE-*Tghmt* and ∆*Tghmt*). **B** Correlation matrix analysis of transcriptomes of strains and their repeats. There was a great correlation between OE-*Tghmt* and ∆*Tghmt* (0.9942 and 0.9921). **C** Venn diagram showed the number of differentially expressed genes, significantly (*P* < 0.05) upregulated or downregulated in hyphae of strains OE-*TgcysK*, ∆*TgcysK*, OE-*Tghmt,* and ∆*Tghmt*, grown on MM + straw at 28 °C for 3 days. Data were obtained by comparison with WT, grown in the same condition. **D** Distribution of significantly (*P* < 0.05) up/down-regulated genes of OE-*TgcysK* relative to WT. **E** KEGG pathway classification on significantly (*P* < 0.05) upregulated genes of OE-*TgcysK* relative to WT, note that genes belonged to amino acid metabolism were up-regulated most, and other up-regulated genes were mainly distributed in energy, carbohydrate, and transport metabolism. **F** Gene ontology classification for significantly (*P* < 0.05) upregulated genes of OE-*TgcysK* relative to WT

PCA results showed that transcriptome of OE-*TgcysK* was significantly different from WT, while there was no significant difference between ∆*TgcysK*, OE-*Tghmt*, ∆*Tghmt*, and good repeatability of the experiment (Fig. 3A). The correlation coefficient matrix showed the correlation between different samples, among which ∆*TgcysK* exhibited the most significant difference to WT (Fig. 3B). Venn showed the numbers of differentially expressed genes in treatments (Fig. 3C). There was no significant difference between transcriptome of OE-*Tghmt* and ∆*Tghmt* and have not significantly up/down-regulated functional genes in OE-*Tghmt* by comparing with WT (Additional file 3:, Additional file 4: Dataset 1B). Thus, Met may not play a key role in the sulfur-promoted lignocellulolytic response, and the phenotype and mechanism of OE-*TgcysK* and ∆*TgcysK* were primarily analyzed in subsequent studies.

The differentially expressed genes in OE-*TgcysK* were mainly ± 4 FoldChanges relative to WT (Fig. 3D). KEGG enrichment showed that the differential genes in OE-*TgcysK* mainly belonged to amino acid metabolism, carbohydrate metabolism, energy metabolism, and transport metabolism (Fig. 3E), which indicated that the increase of intracellular Cys content would affect these processes. Additionally, GO classification from three categories of cell component (CC), molecular function (MF), and biological process (BP) showed the functional distribution of up-regulated genes in OE-*TgcysK* (Fig. 3F). The up-regulation folds of *TgcysK* in transcriptome (10.00 folds) differed from qPCR result (52.35 folds), which might be due to the large gene expression basal values (23,472.7 reads) and fluctuations of transcription level. *TgcysK* was almost undetectable in ∆*TgcysK* and only had 0.9 reads (Additional file 3: Dataset 1A).

The highest up-regulated gene was identified as a membrane sugar transporter belonging to the major facilitator superfamily (MFS) and named *Tgmst*1 (GENE ID: A1A101071.1, NCBI ID: M431DRAFT_463245). *Tgmst*1 was up-regulated 36.3 folds in OE-*TgcysK* and down-regulated 3.3 folds in ∆*TgcysK*. Interestingly, a homofunctional gene named *Tgmst*2 with a large FoldChange was also up-regulated (11.7 folds) in OE-*TgcysK* (GENE ID: A1A110058.1, NCBI ID: M431DRAFT_70791). *Tgmst*2 was also related to transmembrane transport and belonged to MFS (Additional file 3: Dataset 1A). These results signified that an increase in intracellular Cys will induce an up-regulation of sugar transportation-related genes, which implied that Cys may strengthen the lignocellulolytic response of NJAU4742 by activating various sugar transporters.

**Fig. 4:**
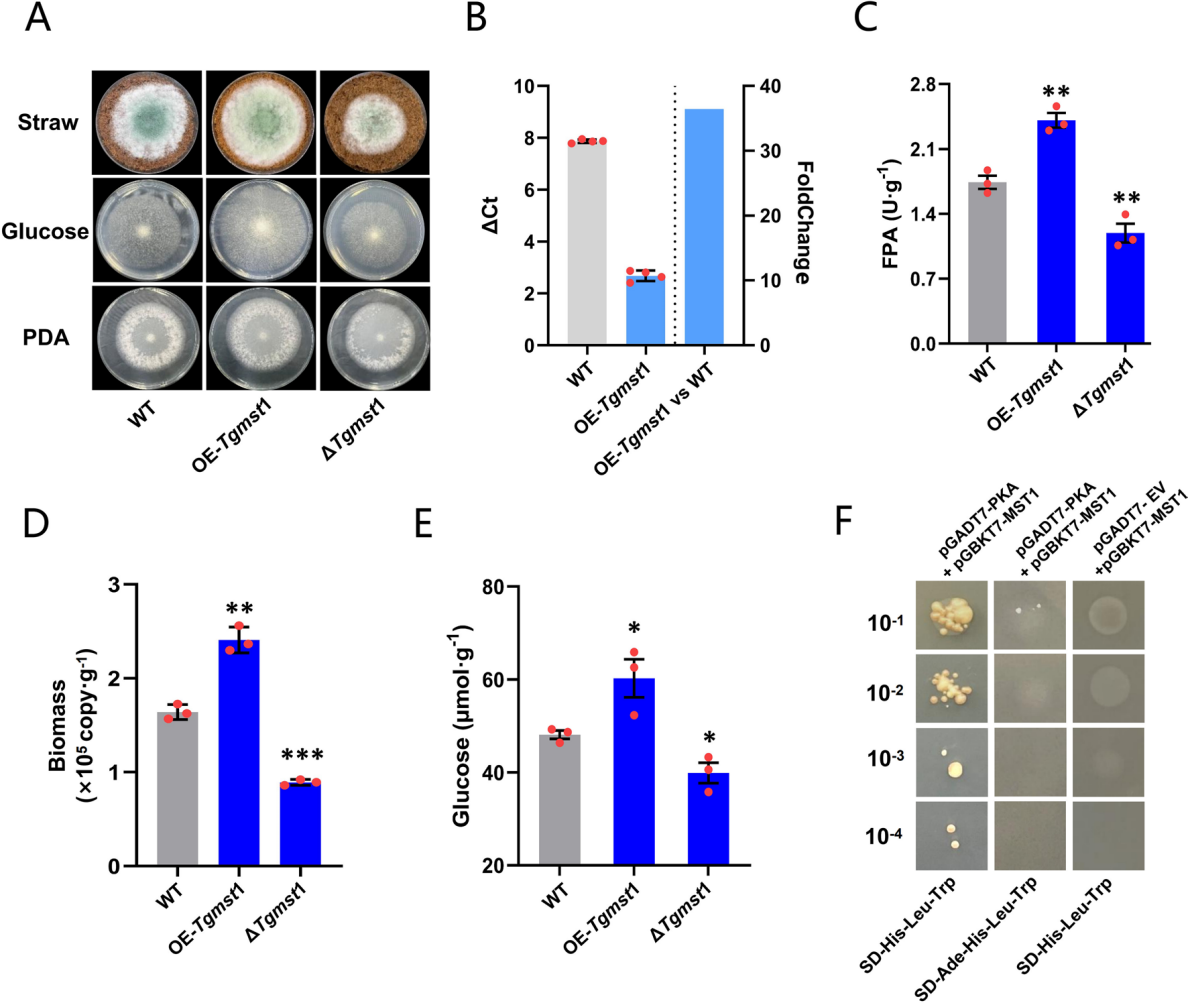
Effect of *Tgmst*1 on lignocellulolytic response and intracellular glucose content of NJAU4742. **A** The growth status of strains (WT, OE-*Tgmst*1, and ∆*Tgmst*1) on MM + straw, MM + glucose, and PDA, grown at 28 °C for 4 days, note that growth of strains on PDA has not obviously different, but there were different on MM + straw and MM + glucose. **B** Relative qPCR results of *Tgmst*1 in OE-*Tgmst*1, FoldChange was obtained by comparison with WT, *Tgmst*1 was 36.44 folds up-regulated in OE-*Tgmst*1. **C** FPA of strains (WT, OE-*Tgmst*1, and ∆*Tgmst*1) on MM + straw, grown at 28 °C 4 days, overexpression of *Tgmst*1 increased enzyme activity, while deletion leads to a decrease. **D** Hyphae biomass of strains (WT, OE-*Tgmst*1, and ∆*Tgmst*1)**,** grown on MM + straw at 28 °C for 4 days. **E** The intracellular glucose content of strains (WT, OE-*Tgmst*1, and ∆*Tgmst*1), and hyphae were washed and ground, and glucose was extracted with buffer and determined. overexpression of *Tgmst*1 increased the intracellular glucose content. **F** Y2H assay indicated the strong interaction between MST1 and PKA, see Additional file 1: Fig. S6C for the original image. pGADT7-EV and pGBKT7-MST1 were transformed to verify the self-activate of MST1 in yeast, and the transformant cannot grow on SD-His-Leu-Trp; pGADT7-PKA and pGBKT7-MST1 were transformed to verify the interaction between MST1 and PKA, and the transformant can grow normally on SD-His-Leu-Trp. The yeast with a 10^–1^ dilution ratio. SD (-Ade)-His-Leu-Trp is SD medium without (Adenine) Histidine, Leucine, and Tryptophan. Bars represent mean ± SEM, with n = 3 biological repeats; red dots resemble values from individual experiments. Student’s *t*-testing was conducted in (**C**, **D**, **E**), **significant difference to WT at two-tailed *P* = 0.0084 (**C**, OE-*Tgmst*1), 0.0043 (**B**, ∆*Tgmst*1); **significant difference to WT at two-tailed *P* = 0.0032 (**C**, OE-*Tgmst*1), ***significant difference to WT at two-tailed *P* = 0.00015 (**C**, ∆*Tgmst*1); *significant difference to WT at two-tailed *P* = 0.047 (**E**, OE-*Tgmst*1), 0.025 (**E**, ∆*Tgmst*1)

Up-regulation of MST1 caused an increase in intracellular glucose content. Since *Tgmst*1 and *Tgmst*2 belong to the same family and the expression base level and FoldChange of *Tgmst*1 are much larger than *Tgmst*2, *Tgmst*1 was selected as the primary gene for research. The *Tgmst*1 overexpression strain OE-*Tgmst*1 (36.44 folds up-regulated) (Fig. 4B) and *Tgmst*1 deletion mutant ∆*Tgmst*1 were constructed to reveal the function of MST1 in NJAU4742. The growth of OE-*Tgmst*1 was better than WT on MM + straw and MM + glucose, while ∆*Tgmst*1 grew worse than WT (Fig. 4A). FPA of OE-*Tgmst*1 and ∆*Tgmst*1 were 2.3 U g^−1^ and 1.2 U g^−1^, respectively, and WT was 1.7 U g^−1^ (Fig. 4C); EG, CBH, and Xylanase activities of OE-*Tgmst* were significantly higher than that of WT (Additional file 1: Fig. S5D). Similarly, the biomass (2.41 × 10^5^ copy g^−1^) and intracellular glucose content (58.2 µmol g^−1^) of OE-*Tgmst*1 were both higher than that of WT (1.64 × 10^5^ copy g^−1^, 47.2 µmol g^−1^). The biomass and intracellular glucose of ∆*Tgmst*1 (0.89 × 10^5^ copy g^−1^ and 38.9 µmol g^−1^) were significantly lower than that of WT, which implied that MST1 is a critical sugar transporter for NJAU4742, and its absence dramatically affects sugar transport capacity, even on the PDA (Fig. 4A, D, E). MST1 also shared high homology with many glucose/xylose/hexose transporters (Additional file 1: Fig. S10). The results showed that the intracellular glucose content of NJAU4742 was positively correlated with the expression level of *Tgmst*1, which further illustrated that MST1 may be responsible for glucose transportation.

GPCR/cAMP/PKA was a conserved extracellular glucose recognition and signals transduction pathway, and PKA was a conservative protein [14]. The vectors pGADT7-PKA and pGBKT7-MST1 were constructed to perform the yeast two-hybrid assay (Y2H). Yeast transformant grew normally on SD-His-Leu-Trp medium, which suggested that PKA and MST1 have protein–protein interaction. Concurrently, it was almost unable to grow on the SD-Ade-His-Leu-Trp medium, indicating the relatively weak interaction between PKA and MST1 (Fig. 4F). These results obtained above further certify that MST1 was a sugar transporter depending on the activation of PKA.

### The optical glucose FRET sensor further verified the function of MST1

The strain *Tg*GluSensor expressing the optical glucose FRET sensor was constructed to monitor the intracellular glucose content in living cells. Since repeated sequences in genome will induce DNA repair, the sequence of the FRET sensor was inserted into the genome while removing the bio-marker (*hph*, *ura*3) (Additional file 1: Fig. S2C). The FRET sensor gene was cloned from pDR-GW FLII12Pglu-700μ∆6 (Addgene plasmid #28,002). The fluorescence of FRET sensor comes from cyan fluorescent protein (CFP) and yellow fluorescent protein (YFP), both of which were linked via glucose recognition subunit MglB. Glucose binding will change the conformational of MglB, which leads to a change in distance or orientation between CFP and YFP, resulting in a FRET change (Additional file 1: Fig. S2B). The fluorescence intensity ratio of YFP and CFP (*F*_Y_/*F*_C_) could characterize the intracellular glucose content, and the *F*_Y_/*F*_C_ value was positively correlated with intracellular glucose content [24, 25].

**Fig. 5:**
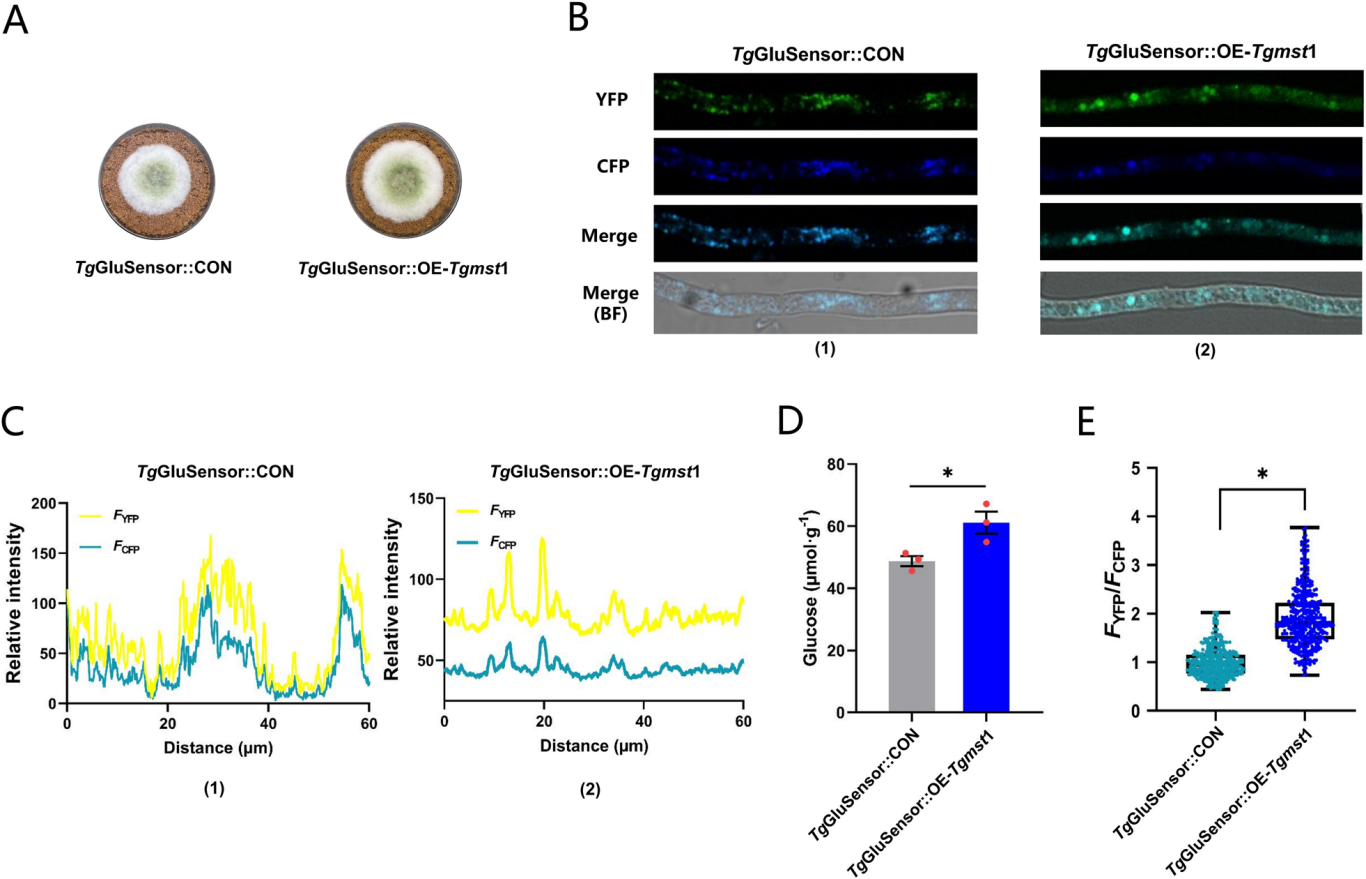
The Optical glucose FRET sensor further indicated that MST1 facilitated the increase of intracellular glucose. **A** Growth status of *Tg*GluSensor::CON and *Tg*GluSensor::OE-*Tgmst*1 on MM + straw, grown at 28 °C for 4 days. **B** The newly grown hyphae of *Tg*GluSensor::CON and *Tg*GluSensor::OE-*Tgmst*1 were gently scraped by glass. *F*_Y_ and *F*_C_ were observed by a CLSM. **B1, 2** Fluorescence images of *Tg*GluSensor::CON and *Tg*GluSensor::OE-*Tgmst*1. YFP was represented by green, and CFP was represented by blue, which was helpful for differences observation. The top one was emitting light of YFP (green), the second one was emitting light of CFP (blue), the third was a merged image of YFP and CFP, and the bottom was a merged image of YFP and CFP in white light. YFP and CFP were excited at 500 nm and 430 nm respectively and received at 525 nm and 470 nm respectively; hyphae length in visual field was 60 µm; **C** Relative fluorescence intensity of hyphae was calculated by the software (ZEN3.4). **D** The intracellular glucose content of *Tg*GluSensor::CON and *Tg*GluSensor::OE-*Tgmst*1, which were grown at 28 °C for 4 days. **E** The mean value of *F*_Y_/*F*_C_ of *Tg*GluSensor::CON (0.89) and *Tg*GluSensor::OE-*Tgmst*1 *(*1.66) were obtained by counting pixel fluorescence intensity. Bars represent mean ± SEM, with n = 3 biological repeats; red dots resemble values from individual experiments. Student’s *t*-testing was conducted in (**D**, **E**), *significant difference to *Tg*GluSensor::CON at two-tailed *P* = 0.027 (**D**, *Tg*GluSensor::OE-*Tgmst*1); *significant difference to *Tg*GluSensor::CON at two-tailed *P* = 0.018 (**E**, *Tg*GluSensor::OE-*Tgmst*1)

The strain *Tg*GluSensor was used as original strain and the *Tgmst*1 overexpression strain *Tg*GluSensor::OE-*Tgmst*1 was constructed. Meanwhile, the *ura*3 gene was backfilled into the *Tg*GluSensor genome to obtain the control strain *Tg*GluSensor::CON. *Tg*GluSensor::OE-*Tgmst*1 and *Tg*GluSensor::CON were cultured on MM + straw (Fig. 5A). The intracellular glucose content of *Tg*GluSensor::OE-*Tgmst*1 (61.1 µmol g^−1^) was significantly higher than that of *Tg*GluSensor::CON (48.6 µmol g^−1^) (Fig. 5D). The fluorescence intensity detected by confocal laser scanning microscope (ZEISS LSM 980), and the *F*_Y_/*F*_C_ ratio was counted by ZEN3.4 (Fig. 5B1, 2). The *F*_Y_ of *Tg*GluSensor::OE-*Tgmst*1 was significantly higher than the *F*_C_, while the *F*_Y_ of *Tg*GluSensor::CON was almost the same as the *F*_C_ (Fig. 5C1, 2). The *F*_Y_/*F*_C_ ratio was calculated by counting the pixel luminous intensity. The *F*_Y_/*F*_C_ median of *Tg*GluSensor::OE-*Tgmst*1 and *Tg*GluSensor::CON were1.66 and 0.89 (Fig. 5E) respectively, which indicated that the intracellular glucose concentration of *Tg*GluSensor::OE-*Tgmst*1 was significantly higher than that of *Tg*GluSensor::CON in living cell. These results further support that MST1 was a sugar transporter with glucose transport capacity.

### GRP regulated the transcription of *Tgmst*1 and *Tgmst*2 by interacting with their promoters

**Fig. 6:**
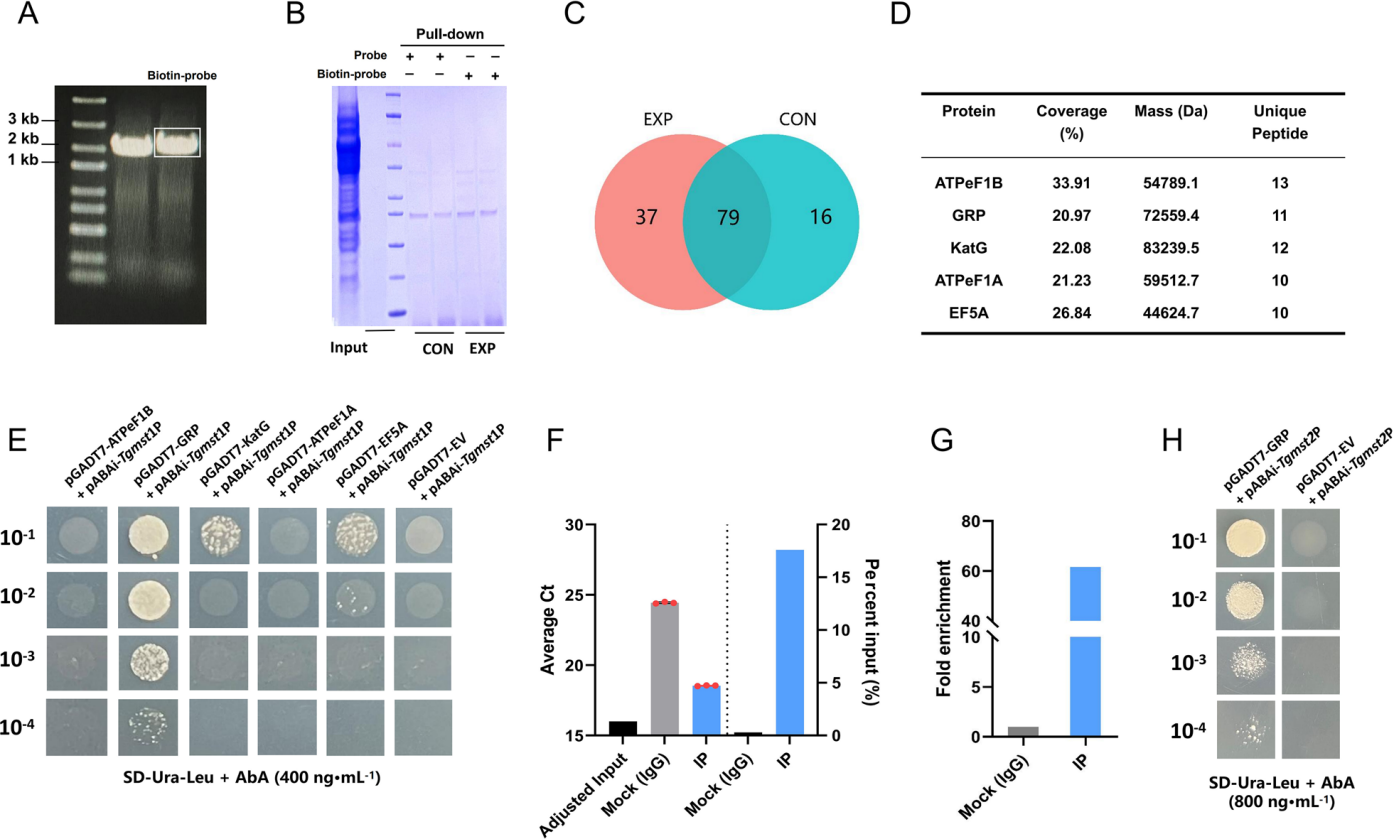
DNA pull-down assay screened the transcription regulator of *Tgmst*1. **A** 2 kb upstream of *Tgmst*1 was amplified as a probe by primers and biotin-primers respectively. Note that the products of biotin primers (right) were slightly larger than those of normal primers (left). **B** Pulled-down proteins were separated by SDS-PAGE and stained by Coomassie brilliant blue (R250). **C** Venn showed the number of differential/common identified proteins in EXP and CON. 79 common proteins and 37 differential proteins of EXP. **D** 37 differential proteins were ranked according to mass spectrum signal intensity. Low signal intensity and substance synthase were filtered, and 5 proteins were considered as the possible transcription factors of *Tgmst*1*.* **E** DNA–Protein interaction between the 5 proteins and *Tgmst*1 promoter was verified by Y1H assay, see Additional file 1: Fig. S6A for original image. Bait-reporter could not grow in SD-Ura medium containing AbA (400 ng mL^−1^). Victors pGADT7-ATPeF1B pGADT7-GRP, pGADT7-KatG, pGADT7-ATPeF1A, pGADT7-EF5A, and pGADT7-EV were respectively transformed into the bait-reporter. GRP and *Tgmst*1 promoter have strong interaction; ATPeF1B and ATPeF1A have no interaction with *Tgmst*1 promoter; KatG and EF5A have weak interaction with *Tgmst*1 promoter. **(F)** ChIP-qPCR assay verified the interaction between GRP and *Tgmst*1 promoter. DNA obtained by immunoprecipitation was detected by qPCR. Percent Input was counted, *Tgmst*1 promoter accounts for 17.6% in IP, 0.296% in Mock (IgG); **(G)** Fold enrichment was counted, and the immune coprecipitation enrichment folds of IP was 61.6 folds relative to Mock (IgG). **H** Y1H assay of *Tgmst*2 promoter region and GRP, see Additional file 1: Fig. S6B for original image; Bait-reporter (pGADT7-EV + pABAi-*Tgmst*2P) could not grow in SD medium containing AbA (800 ng mL^−1^); the transformant (pGADT7-GRP + pABAi-*Tgmst*2P) could grow in the SD medium containing AbA (800 ng mL^−1^)

DNA pull-down assay was performed to confirm the transcription factor of *Tgmst*1. The promoter region (− 2 kb to − 1 bp) of *Tgmst*1 was amplified by a biotin-labeled primer (Additional file 1: Fig. S3A) and then combined with streptavidin magnetic beads as the probe (EXP); the fragment without biotin was amplified to be used as control (CON) (Fig. 6A). The probe was incubated with nucleoprotein, and magnetic beads were used to pull down the probe-bound proteins. The precipitated proteins were separated by SDS-PAGE to detect differences between EXP and CON (Fig. 6B). Differential protein strips were digested into polypeptides and identified by LC–MS/MS. Raw data was submitted to the ProteinPilot for NJAU4742 genome database retrieval analysis. With confidence ≥ 0.95 and unique peptides ≥ 1, the numbers of secondary mass spectra peaks of EXP and CON were 431 and 339, and the identified protein numbers of EXP and CON were 116 and 95, while the shared protein numbers were 79 (Fig. 6C). IPR analysis results showed that a high abundance of Hsp70 structural domains were detected in pull-down products (Additional file 1: Fig. S3B), and GO and KEGG enrichment revealed the proteins functions and their associated metabolic pathways (Additional file 1: Fig. S3C, D). The identified proteins were scored according to the signal intensity of mass spectrometry. Proteins with high scores were listed as follows: F-type H^+^-transporting ATPase subunit β (ATPeF1B, GENE ID: A1A106337.1, NCBI ID: KKP03928.1), glucose regulation-related protein (GRP, GENE ID: A1A105631.1, NCBI ID: KKP00680.1), catalase-peroxidase (KatG, GENE ID: A1A103615.1, NCBI ID: OPB43868.1), F-type H^+^-transporting ATPase subunit α (ATPeF1A, GENE ID: A1A100553.1, NCBI ID: OPB46749.1), and elongation factor 5α (EF5A, GENE ID: A1A106315.1, NCBI ID: OPB41649.1) (Fig. 6D).

The interactions between these five proteins and the promoter of *Tgmst*1 were evaluated through yeast one-hybrid assay (Y1H). The vector pAbAi-*Tgmst*1P was constructed by inserting the *Tgmst*1 promoter fragment (− 500 to − 1 bp) and it was transformed into Matchmaker Gold Yeast. The transformant (Bait-reporter) cannot grow on SD-Ura containing 400 ng mL^−1^ aureobasidin A (AbA). Subsequently, the vectors pGADT7-ATPeF1B, pGADT7-GRP, pGADT7-KatG, pGADT7-ATPeF1A, and pGADT7-EF5A were constructed and transformed respectively into bait-reporter. The results indicated that there was a strong interaction between GRP and *Tgmst*1 promoter, and both KatG and EF5A could weakly interact with *Tgmst*1 promoter, while ATPeF1A and ATPeF1B barely interacted with *Tgmst*1 promoter (Fig. 6E).

Furthermore, ChIP-qPCR assay was performed to verify the interaction between GRP and *Tgmst*1 promoter. The strain GRP-His that 6 × His-tag was added to the C-terminus of GRP was constructed and verified by Western blot (Additional file 1: Fig. S4A). After cross-linking and nucleic acid breaking, GRP-binding DNA fragments were precipitated by a ChIP-class antibody, and the DNA combined by GRP was collected. qPCR was respectively carried out by using the collected DNA of IP, Mock (IgG), and Input as the template to detect the copies of *Tgmst*1 promoter fragment. The Cts values from primers P-5 and P-6 were significantly different from IP and Mock (IgG) (Additional file 1: Fig. S4B, C1). The qPCR results from primer P-6 were used to count Percent input (Additional file 1: Fig. S4C2) and Fold enrichment (Additional file 1: Fig. S4C3). The Percent input of IP and Mock (IgG) was 17.6% and 0.296%, respectively (Fig. 6F). The Fold enrichment could indicate the specific binding efficiency, and results showed that the enrichment efficiency of GRP for *Tgmst*1 promoter in IP was 61.6 folds higher than that in Mock (IgG) (Fig. 6G). The amplification range of primer P-6 was -363 bp to -224 bp, which probably contained the binding region of GRP to *Tgmst*1 promoter.

In addition, we were curious about whether another up-regulated MFS protein gene *Tgmst*2 was also regulated by GRP protein. The DNA–protein interaction verification between GRP and *Tgmst*2 promoter (− 500 to − 1 bp) was also conducted through Y1H assay. Surprisingly, GRP and *Tgmst*2 promotor also exhibited interaction (Fig. 6H), which indicated that the up-regulation of *Tgmst*2 might also be achieved through GRP.

### *Tgmst*1 and *Tgmst*2 were positively transcriptional regulated by GRP

**Fig. 7:**
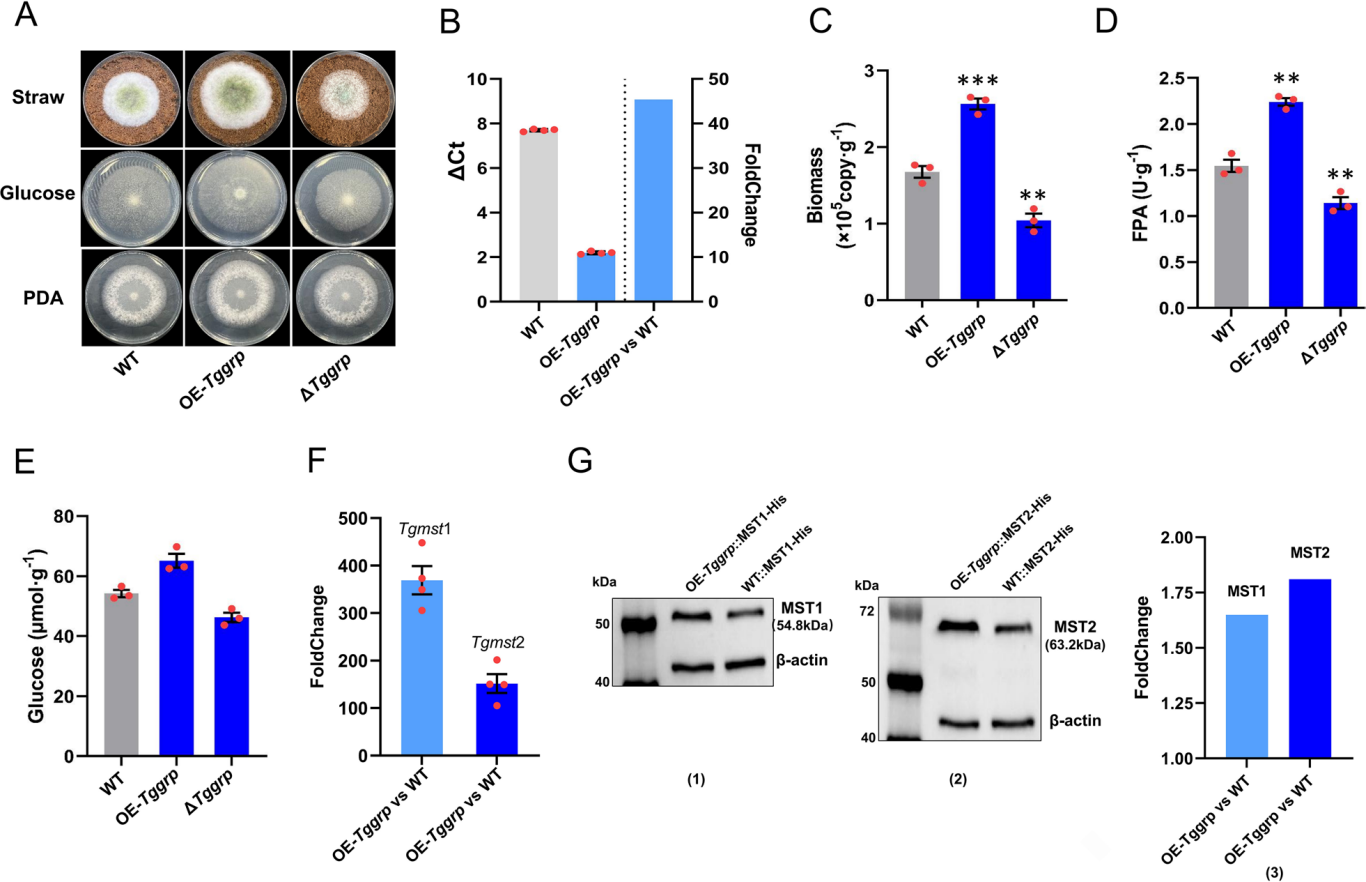
Function of *Tggrp* on lignocellulolytic response and transcription of *Tgmst*1 and *Tgmst*2. **A** The growth status of strains (WT, OE-*Tggrp*, and ∆*Tggrp*) on MM + straw, MM + glucose, and PDA, grown at 28 °C for 3 days, note that growth of strains was not different in PDA, but there were differences on MM + straw and MM + glucose. **B** FoldChange of OE-*Tggrp* relative to WT, 45.41 folds up-regulation in OE-*Tggrp*. **C** Hyphae biomass of strains (WT, OE-*Tggrp*, and ∆*Tggrp*), grown on MM + straw at 28 °C for 4 days. **D** FPA of strains (WT, OE-*Tggrp*, and ∆*Tggrp*) on MM + straw, grown at 28 °C for 4 days. Overexpression of *Tggrp* enhanced enzyme activity, while deletion led to a decrease. **E** The intracellular glucose content of strains (WT, OE-*Tggrp*, and ∆*Tggrp*). overexpression of *Tgmst*1 increased intracellular glucose content. **F** FoldChange of *Tgmst*1 in OE-*Tggrp* and ∆*Tggrp*. *Tgmst*1 and *Tgmst*2 were up-regulated by 369.1 folds and 151.7 folds respectively in OE-*Tggrp*. **G** Western blot of MST1 and MST2 in OE-*Tggrp* and WT, see Additional file 1: Fig. S6D for original image. **G1, 2** Western blot of MST1/2 andβ-actin. **G3** FoldChange of MST1 and MST2 in OE-*Tggrp*, MST1 and MST2 were respectively up-regulated 1.7 and 1.8 folds in OE-*Tggrp*. Bars represent mean ± SEM, with n = 3 biological repeats; red dots resemble values from individual experiments. Student’s *t*-testing was conducted in (**C**, **D**, **E**), ***significant difference to WT at two-tailed *P* = 0.00046 (**C**, OE-*Tggrp*), **significant difference to WT at two-tailed *P* = 0.0028 (**C**, ∆*Tggrp*); **significant difference to WT at two-tailed *P* = 0.0012 (**D**, OE-*Tggrp*), 0.0086 (**D**, ∆*Tggrp*); *significant difference to WT at two-tailed *P* = 0.011 (**E**, OE-*Tggrp*), 0.043 (**E**, ∆*Tggrp*)

The interaction between GRP and *Tgmst*1 promoter might be accompanied by a specific regulatory relationship. To verify this hypothesis, *Tggrp* deletion mutant ∆*Tggrp* and *Tggrp* overexpression strain OE-*Tggrp* (45.4 folds up-regulated, Fig. 7B) were constructed. OE-*Tggrp* grew better on MM + straw than WT, and ∆*Tggrp* was worse than WT, and they did not differ in growth on PDA. It was noteworthy that the editing of *Tggrp* affected the growth on MM + glucose, suggesting that *Tggrp* might be able to influence glucose acquisition (Fig. 7A). The biomass (2.56 × 10^5^ copy g^−1^) and intracellular glucose content (65.2 µmol g^−1^) of OE-*Tggrp* were both higher than WT (1.68 × 10^5^ copy g^−1^, 54.2 µmol g^−1^), and these parameters were declined in ∆*Tggrp* (1.04 × 10^5^ copy g^−1^ and 46.3 µmol g^−1^) (Fig. 7C, E). The FPA of OE-*Tggrp* and ∆*Tggrp* were 2.24 U g^−1^ and 1.14 U g^−1^, respectively, and it was 1.55 U g^−1^ of WT (Fig. 7D). Similarly, the EG, CBH, and Xylanase activities of OE-*Tggrp* were significantly higher than that of WT, while these parameters were dropped in ∆*Tggrp* (Additional file 1: Fig. S5E). The qPCR on *Tgmst*1 and *Tgmst*2 was conducted, and the expression level of *Tgmst*1 and *Tgmst*2 were up-regulated by 369.1 folds and 151.7 folds respectively in OE-*Tggrp* (Fig. 7F), which indicated that GRP owned a positive regulation to *Tgmst*1 and *Tgmst*2.

To demonstrate the concordance between protein level of MST1/2 and transcription level of *Tgmst*1/2, the strains OE-*Tggrp*::MST1/2-His and WT::MST1/2-His were constructed by adding 6 × His-tag to the C-terminus of MST1/2 based on OE-*Tggrp* and WT, respectively. The expression level of MST1 and MST2 in OE-*Tggrp* and WT was determined by Western blot, and β-actin as the internal reference. The results indicated that MST1 and MST2 were respectively up-regulated by 1.7 folds and 1.8 folds in OE-*Tggrp* (Fig. 7G), which suggested that the protein expression level of MST1 and MST2 was consistent with transcription level in OE-*Tggrp*.

### GRP could affect the intracellular glucose content of NJAU4742

**Fig. 8:**
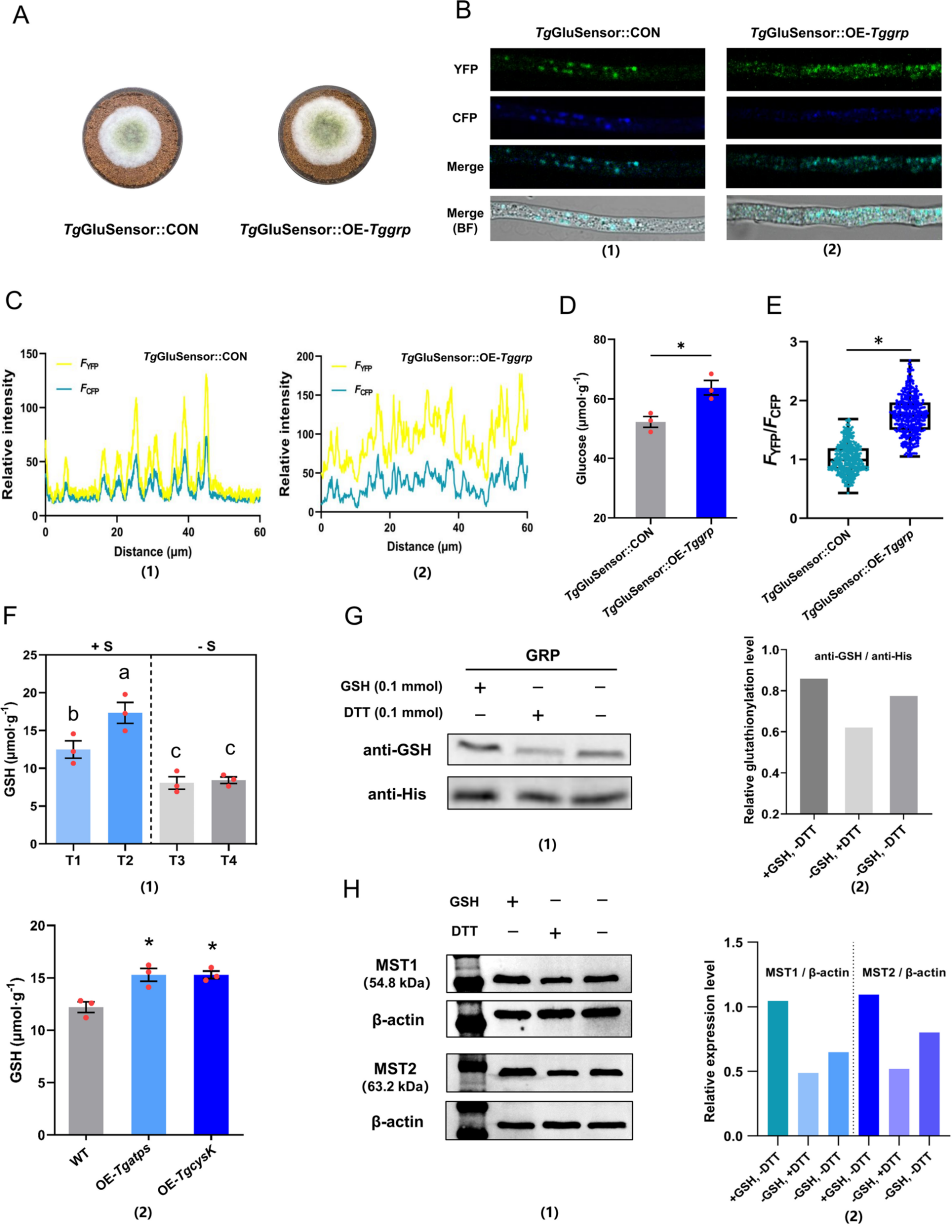
Mechanism of GRP regulating the expression of MST1 and MST2. **A** Growth states of *Tg*GluSensor::CON and *Tg*GluSensor::OE-*Tggrp* on MM + straw, grown at 28 °C for 4 days. **B** Fluorescence images of *Tg*GluSensor::CON and *Tg*GluSensor::OE-*Tggrp*. The newly grown hyphae of *Tg*GluSensor::CON and *Tg*GluSensor::OE-*Tggrp* were gently scraped by glass, *F*_Y_ and *F*_C_ were observed by a CLSM. Note that *F*_Y_ and *F*_C_ were same in *Tg*GluSensor::CON and *F*_Y_ was brighter than *F*_C_ in *Tg*GluSensor::OE-*Tggrp*. YFP and CFP were excited at 500 nm and 430 nm and received at 525 nm and 470 nm respectively; hyphae length in visual field was 60 µm. **C** Relative fluorescence intensity of hyphae calculated by software (ZEN3.4). **D** The intracellular glucose content of *Tg*GluSensor::CON and *Tg*GluSensor::OE-*Tggrp*, grown for 4 days at 28 °C. **E** The median *F*_Y_/*F*_C_ value of *Tg*GluSensor::CON and *Tg*GluSensor::OE-*Tggrp*. **F1** GSH content of treatments (T1, T2, T3, and T4). **F2** GSH content of OE-*Tgatps* and OE-*TgcysK*. **G** Western blot verified that GSH or DTT could affect the glutathionylation level of GRP; (**g1**) 1:500 anti-GSH antibody was used in Western blot; GSH (0.1 mmol) or DTT (0.1 mmol) was added to MM + straw, “ + ” means added, “−” means not added. With same Input, the hybridization signal intensity of anti-GSH antibody was increased in GSH added treatment (+ GSH, − DTT), and decreased in DTT added treatment (− GSH, + DTT). **g2** The glutathionylation level of GRP in different treatments. **H** The effect of different glutathionylation levels of GRP on MST1/2 expression level, see Additional file 1: Fig. S6E for original image. (h1) 1:1000 anti-6 × His antibody was used in Western blot; GSH (0.1 mmol) or DTT (0.1 mmol) was added to MM + straw; the hybridization signal intensity of anti-6 × His antibody was increased in GSH added treatment (+ GSH, − DTT), and decreased in DTT added treatment (− GSH, + DTT). (h2) The expression level of MST1/2 in different treatment. Bars represent mean ± SEM, with n = 3 biological repeats; red dots resemble values from individual experiments. Student’s *t*-testing was conducted in (**D**, **E**), *significant difference to *Tg*GluSensor::CON at two-tailed *P* = 0.015 (**D**, *Tg*GluSensor::OE-*Tggrp*); *significant difference to *Tg*GluSensor::CON at two-tailed *P* = 0.031 (**E**, *Tg*GluSensor::OE-*Tggrp*). ANOVA was conducted in (**F1**), Tukey's HSD test was used for post hoc comparisons, and the letters “a”, “b”, and “c” were used for significance exhibition. Inorganic sulfide has a significant effect on the intracellular GSH content (*P* < 0.05 level). Student’s *t*-testing was conducted in (**F2**), *significant difference to WT at two-tailed *P* = 0.012 (**F2**, OE-*Tgatps*), 0.012 (**F2**, OE*-TgcysK*)

To prove that GRP could affect the intracellular glucose content by regulating the expression of MST1 and MST2, *Tggrp* overexpression strain *Tg*GluSensor::OE-*Tggrp* was constructed based on the strain *Tg*GluSensor. After growing on MM + straw at 28 ℃ for 4 days (Fig. 8A), the intracellular glucose concentration of *Tg*GluSensor::OE-*Tggrp* (63.8 µmol g^−1^) was significantly higher than that of *Tg*GluSensor::CON (52.3 µmol g^−1^) (Fig. 8D). Simultaneously, the *F*_Y_ was significantly higher than *F*_C_ in *Tg*GluSensor::OE-*Tggrp*, while *F*_Y_ was almost similar to *F*_C_ in *Tg*GluSensor::CON (Fig. 8B). The statistical results of fluorescence intensity were also consistent with the results of graph (Fig. 8C). The median of *F*_Y_/*F*_C_ ratio in *Tg*GluSensor::OE-*Tggrp* was 1.72, and it was 0.98 in *Tg*Glu::Sensor-CON (Fig. 8E), which indicated that the intracellular glucose concentration of *Tg*GluSensor::OE-*Tggrp* was higher than that of *Tg*GluSensor::CON in living cell. The results showed that GRP could also promote glucose transportation by up-regulating the expression of *Tgmst*1 and *Tgmst*2.

### Sulfide addition facilitated GSH synthesis and induced glutathionylation of GRP

Inorganic sulfide assimilation could produce large amounts of primary assimilate Cys (Additional file 1: Fig. S1A), which in turn facilitated the synthesis of glutathione (GSH). Metabolomic analysis (T1 and T3) showed that GSH metabolism was one of major metabolisms that dramatically responded to increased exogenous inorganic sulfide, and the GSH content in T3 was higher than that in T1 (Additional file 1: Fig. S7, Additional file 7: Dataset 3 line5). GSH could induce glutathionylation by forming the disulfide bonds with cysteine residues of proteins [26]. The glutathionylation of transcription factors was beneficial to maintain the structural stability of functional domains and promote the efficiency of DNA binding domain or activation domain, thus increasing the transcriptional activation efficiency [27].

The intracellular GSH content of WT growing in four treatments (T1, T2, T3, and T4) and the two strains (OE-*Tgatps* and OE-*TgcysK*) were determined. The intracellular GSH contents in T1 (12.5 µmol g^−1^) and T2 (17.3 µmol g^−1^) were both significantly higher than that in T3 (7.6 µmol g^−1^) and T4 (8.1 µmol g^−1^) (Fig. 8F1). The intracellular GSH contents in OE-*Tgatps* (15.4 µmol g^−1^) and OE-*TgcysK* (15.3 µmol g^−1^) were also significantly higher than that of WT (12.2 µmol g^−1^) (Fig. 8F2). These results further illustrated that exogenous addition with inorganic sulfide or enhancing the sulfur assimilation would increase the intracellular GSH content.

Glutathionylation is common in Hsp70 family proteins and has positive implications for stabilizing protein structure and promoting substrate catalytic efficiency. Generally, Hsp70 homologs have two individual domains, the nucleotide-binding domain (NBD) and the substrate-binding domain (SBD), which were connected by a flexible linker [28, 29]. GRP, a typical Hsp70 family protein, was also likely to be glutathionylated when intracellular GSH levels were elevated. Commercial anti-GSH antibodies were usually used to evaluate the glutathionylation level. However, the currently available anti-GSH antibody could only test the purified protein [30]. Here, the strain GRP-His was cultured in T1 conditions for protein extraction. GRP was purified by nickel ion magnetic beads for Western blotting. The rabbit polyclonal anti-GSH antibody was primary antibody, and Alexa Flour 488 labeled goat anti-rabbit IgG (H + L) antibody as secondary antibody. The antibody hybridization signal was detected (Additional file 1: Fig. S1B), which indicated that the increase in intracellular GSH caused by inorganic sulfide addition could induce the glutathionylation of GRP.

### The expression of MST1 and MST2 positively correlated with glutathionylation level of GRP

Based on the previous results, inorganic sulfide addition could induce glutathionylation of GRP by elevating the intracellular GSH content. Therefore, glutathionylation of GRP might play a central role in the pathway of Cys promoting the up-regulation of *Tgmst*1/2. Exogenous GSH addition could directly improve the glutathionylation level of some proteins, while dithiothreitol (DTT) could break the disulfide bond and prevent its formation [28]. Since MM + straw was a solid medium, it was not available to show the dosage of GSH and DTT in terms of concentration. Thus, 0.1 mmol of GSH and DTT were added to each plate. GRP was isolated and purified and the concentration was equalized. The hybridization signal intensity of anti-GSH antibody was increased in GSH added treatment (+ GSH, − DTT) and decreased in DTT added treatment (− GSH, + DTT) compared to the control without GSH and DTT addition (− GSH, − DTT) (Fig. 8G1). Notably, since treatments have equal inputs, the blot area was consistent, but there was a significant difference in gray-depth of blot. Gray-depth was dependent on the glutathionylation level of GRP. In this study, the relative glutathionylation level of GRP was used to describe the glutathionylation degree. The grayscale of blots in WB images was counted by the software Image J, and the statistics showed that the relative glutathionylation level of GRP in GSH added treatment (+ GSH, − DTT) was higher than control (− GSH, − DTT), while DTT added treatment (− GSH, + DTT) was lower than control (Fig. 8G2). This result suggested that the glutathionylation level of GRP could be regulated by exogenous GSH or DTT addition.

To investigate the effect of different glutathionylation levels on GRP regulating the expression of MST1 and MST2, the strains WT::MST1-His and WT::MST2-His were cultured on MM + straw added with GSH or DTT, after which the total protein of each treatment was extracted and normalized. The expression level of MST1 and MST2 was quantified by Western blot with β-actin as internal reference. Here, both blot gray-depth and area of MST1 and MST2 were significantly increased in GSH added treatment, while the opposite result was obtained in DTT added treatment (Fig. 8H1). By comparing the internal reference adjusted MST1 and MST2 expression levels of treatments, GSH exhibited a promoting effect on MST1 and MST2 expression while DTT inhibited this process (Fig. 8H2). Previous results have shown that GSH induces glutathionylation while DTT induces deglutathionylation, therefore the results shown in Fig. 8H indicated that the expression level of MST1/2 was positively correlated with glutathionylation level of GRP. Therefore, it was reasonable to extrapolate that Cys promoted MST1/2 expression by indirectly increasing the glutathionylation level of GRP, which in turn increased sugar transport efficiency and ultimately enhanced the lignocellulolytic response of NJAU4742.

## Discussion

The bioconversion of straw to alcohol chemicals usually requires saccharification by microorganisms or industrial lignocellulases followed by fermentation by *Saccharomyces* *cerevisiae* [31, 32]. Recombining the fermentation and alcohol tolerance related genes into filamentous fungi is a promising way to achieve a one-step conversion of straw to alcohol or organic acid [33, 34]. Fermentation requires a constant and stable monosaccharides supply, and efficient sugar transport can greatly improve fermentation efficiency. Up-regulation of sugar transport capacity also facilitates efficient cellulase production by microbial cell factories.

Sulfur deposition caused an increase in soil fungi/bacteria ratio, inducing microorganisms to obtain more carbon by increasing the ratio of carbon/nitrogen-acquiring enzymes while decreasing carbon inputs for respiration and increasing carbon utilization efficiency to accommodate carbon limitation [35]. This research enlightened us to find the connection between sulfur assimilation and carbon source utilization pathway. To reveal the response of intracellular metabolites to inorganic sulfur addition, we conducted a metabolomics analysis. Intracellular glutathione and cysteine were revealed to be significantly affected and showed coupled associations with lignocellulolytic response. As the primary sulfur assimilate product, Cys was an important sulfur storage form in cells [36], which implied to us that Cys was the critical metabolite in response to sulfur addition increase.

The intracellular Cys and Met content were increased by genetic manipulation for transcriptomic analysis. Due to the absence of high-fold differentially expressed genes in the OE-*Tghmt* transcriptome, we excluded the possibility that Met played a central role in the promotion effect of inorganic sulfides (Additional file 4: Dataset 1B). *Tgmst*1 and *Tgmst*2 were the key genes responding to the increased intracellular Cys content. MST1 and MST2 were novel sulfur assimilation-related sugar transporters that belonged to the MFS family, and many monosaccharide transporters share high homology with MST1/2, including xylose transporters, glucose transporters, and hexose transporters (Additional file 1: Fig. S10). In addition, the transport substrates of MST1/2 may not be specific, and the substrate diversity of sugar transporters is universal [37]. For example, the glucose transporter protein HxtB in *Aspergillus* *nidulans* also transports xylose [38]. Tr69957 identified by Roberto N. Silva is responsible for transporting mannose, xylose, and cellobiose [39], and another sugar transporter STP1 is involved in transporting cellobiose and glucose [40]. TrSTR1 is able to transport xylose and arabinose [41]. This implies that this MST1/2 may be able to transport a variety of substrates, including glucose and even cellobiose. Interestingly, Cys could significantly decline the blood sugar [42]. After being fed with Cys for eight weeks, the blood sugar content was also significantly lower than that of control [43, 44]. These results suggest that Cys promotes cellular glucose absorption, which seems similar to our results that Cys can promote cell absorption of extracellular sugar. In summary, with straw as the sole carbon source, the up-regulation of straw hydrolysis products (glucose, xylose, cellobiose, etc.) transport capacity undoubtedly facilitates the maintenance of a vigorous cellular metabolism, especially CAZymes metabolism.

The protein-DNA interaction and regulatory relationship between GRP and *Tgmst*1 and *Tgmst*2 have been verified. Transcription factors (TFs) can be functionally divided into two regions: DNA binding domain (BD), which includes helix turn helix (HTH)/helix loop helix (HLH), zinc finger, and basic leucine zipper (bZIP); transcriptional activation domain (AD), which can affect transcription efficiency by directly or indirectly acting on transcription complex [45–47]. GRP belongs to the Hsp70 family and has a complete function in transcriptional activation. Hsp70 family proteins are ancient and conserved that were first found to be related to heat shock response [48]. More studies showed the functional diversity of Hsp proteins, and they participated in various physiological activities, including signal transduction, transcriptional activation, apoptosis, transmembrane transport, and DNA repair [49, 50]. Hsp70 proteins and their homologs have two separate domains, named ATPase or nucleotide-binding domain (NBD) and substrate-binding domain (SBD), and the functional diversity of Hsp70 proteins mainly originates from the SBD [51]. GRP possessed BIP-like ATP and nucleotide-binding domain and HTH/HLT-like nucleotide-binding domain, which collaborated to comprise the functionality integrity.

Glutathionylation is a common post-translational modification of proteins caused by sulfur compounds. It is the process of forming mixed disulfide between glutathione and protein cysteine residues, and it is also the protective mechanism of active sulfhydryl group [27]. GSH residue is an active molecule, which may show a variety of conformations in solution or combination with protein. The affinity of anti-GSH antibodies may be reduced significantly owing to the changes in protein conformation or thiolation environment [52]. Currently, available anti-GSH antibodies can be used for purified protein analysis, so GRP should be purified before detection. Glutathionylation has diverse effects on protein function, some studies suggested that glutathionylation inhibits the protein function of c-Jun and NF-kB [53, 54]. However, more reports are consistent with our study. Peroxynitrite can increase SERCA activity by glutathionylation, the presence of GSH is required for the functioning of recombinant SERCA in phospholipid vesicles, and its Ca^2+^ uptake activity is dependent on peroxynitrite-induced glutathionylation [55]. Our results showed that the up-regulation of glutathionylation of GRP facilitated its transcriptional activation function, which exhibited the promoting effect of glutathionylation on protein function. In addition, glutathionylation of FABP5 at Cys127 promoted its nuclear translocation, glutathionylated FABP5 would enter nucleus and activate transcription of downstream genes [56]. Similar results were obtained in our study, suggesting that glutathionylation promoting protein transcriptional activation efficiency existed.

As a massive element in cells, sulfur has a global impact on cellular metabolism, other assimilates may jointly be involved in this process. Existing studies on the effects of glutathionylation on proteins are not comprehensive. The glutathionylation site and the mechanism of glutathionylation affect the transcriptional activation efficiency of GRP are not elaborated. However, this research still has significance in biomass utilization, the novel sulfur metabolism-regulated sugar transporter proteins provide new targets of molecular cloning for metabolic engineering, and the revealed regulatory pathway of transporters MST1/2 provides the possibility of subsequent gene controllable expression and metabolic modification. During microbial utilization of sugar for alcoholic fermentation or organic acid synthesis, single-cell sugar transport capacity controls the superior limit of fermentation efficiency, and our study may contribute to increasing the superior limit of fermentation efficiency.

## Conclusion

**Fig. 9:**
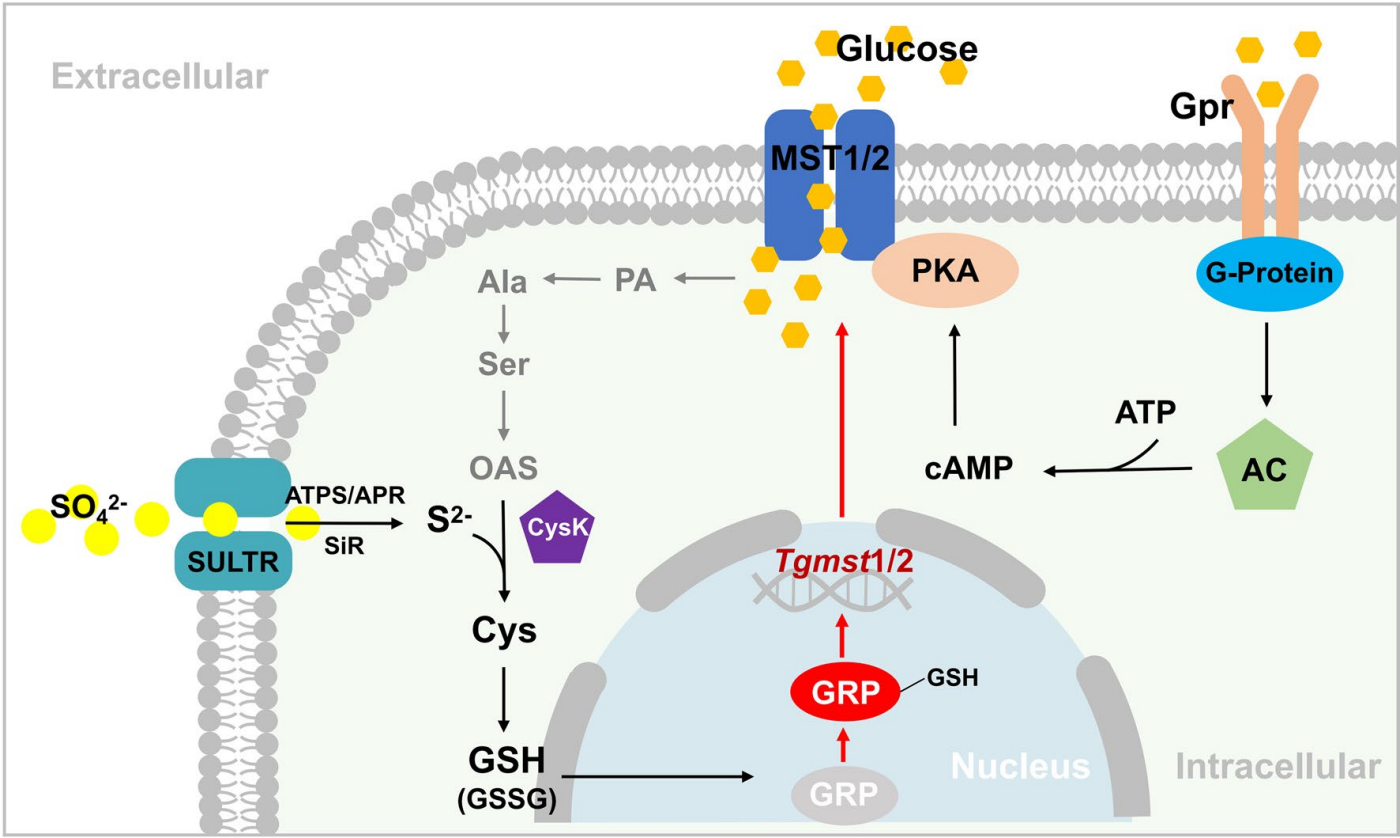
The mechanism of cysteine inducing upregulation of MST1/2 expression. Inorganic sulfur (SO_4_^2−^) was transported into cell by SULTR and reduced into S^2−^ by ATPS/APR/SiR, S^2−^ and OAS synthesized Cys, which is further converted to GSH. The glutathionylation level of GRP would be up-regulated due to increased Intracellular GSH. Glutathionylated GRP could specifically bind the *Tgmst*1/2 promoter and up-regulated MST1/2 expression. Pyruvate and Acetyl-CoA were generated by the Glycolysis and the TCA cycle, which was essential for OAS synthesis, the energy generated by TCA can also be used for sulfur transportation. *PA* pyruvic acid, *Ala* Alanine, *Ser* Serine, *OAS* Acetyl-serine, *Cys* Cysteine, *GSH* Glutathione, *Gpr* Glucose receptor, *G-protein* GTP binding protein, *AC* adenylate cyclase, *PKA* protein kinase A, *ATPS* ATP sulfatase, *APR* APS sulfotransferase, *SiR* Sulfite reductase, *SULTR* Sulfate transporter

This study reported the facilitative effect of inorganic sulfide on lignocellulolytic response of NJAU4742 and elucidated the molecular mechanism of this process (Fig. 9). Cys and GSH were identified as the main differential intracellular metabolites responding to inorganic sulfur addition, and were also closely associated with the lignocellulolytic responses. GSH content was up-regulated due to increased Cys, which induced upregulation of the glutathionylation level of GRP and ultimately upregulated *Tgmst*1/2 expression. The transcriptional level of the *Tgmst*1 and *Tgmst*2 were positively correlated with the glutathionylation level of GRP. MST1 and MST2 were critical proteins that respond to increased intracellular Cys. Up-regulation of MST1 and MST2 enhanced cellular acquisition of extracellular sugar, which facilitated the maintenance of vigorous CAZymes production. In brief, Cys achieved an up-regulation of *Tgmst*1 and *Tgmst*2 by indirectly increasing the glutathionylation level of GRP, which in turn promoted the extracellular sugar transport capacity. Efficient carbon source acquisition enhanced the lignocellulolytic response of NJAU4742.

## Methods

### Plasmids, strains, and culture conditions

Plasmids were propagated in *Escherichia* *coli* DH5α (Tsingke Biotechnology, China). Bacteria were grown in LB medium (tryptone, 10 g L^−1^; yeast extract, 5.0 g L^−1^; NaCl, 5.0 g L^−1^) at 37 °C. *T.* *guizhouense* NJAU4742 (genomic sequence NCBI ID: LVVK00000000.1, Genetic database: https://bioinfo.njau.edu.cn/tgn4742/index.php) was isolated from composting sample and stored in our Lab, growing on PDA/PDB (Oxoid, UK) or MM (Carbon source, 10 g L^−1^; KH_2_PO_4_, 15 g L^−1^; (NH_4_)_2_SO_4_, 10 g L^−1^; MgSO_4_, 0.6 g L^−1^; FeSO_4_, 5.0 mg L^−1^; MnSO_4_, 1.6 mg L^−1^; ZnSO_4_, 1.4 mg L^−1^; CoCl_2_, 2.0 mg L^−1^; CaCl_2_ 1.0 g L^−1^) at 28 °C. MM + straw was prepared by using liquid MM to adjust the moisture content of straw to 75% for the growth comparison. The Matchmaker Gold Yeast and Y2HGold Yeast (Takara, Japan) were respectively used in yeast one-hybrid (Y1H) and yeast two-hybrid (Y2H), which were grown in YPDA medium (Hopebio, China). SD-Leu-Trp medium was used to screen the positive Y2H transformants, SD-His-Leu-Trp and SD-Ade-His-Leu-Trp medium was used for protein–protein interaction validation. SD-Ura-Leu medium was used to screen the positive Y1H transformants, and SD-Ura-Leu + AbA was used for protein-DNA interaction validation. pAbAi and pGADT7 (Takara, Japan) were used for vector construction in Y1H, while pGBKT7 and pGADT7 (Takara, Japan) were used for Y2H. pDR-GW FLII12Pglu-700μ∆6 (Addgene plasmid #28,002) was used as template to clone the optical intracellular glucose FRET sensor gene. pSilent-1 (Miaolingbio, China) was used to construct the vector to interfere with RNA translation.

### CAZymes activity assay

The CAZymes secreted by hyphae were dissolved in NaAc buffer (HAc, 10 mM; NaAc, 100 mM; pH5.5). The EG /Xylanase / FPA activity assay was carried out as follows: 480 μL CMCNa (0.5%, W/V) / 480 μL Xylan (0.5%, W/V) / Whatman filter paper, 500 μL NaAc buffer, and 20 μL crude enzyme solution were mixed, and the reaction system was kept at 50 °C for 20 min, and then 1 mL DNS (NaKC_4_H_4_O_6_, 185 g L^−1^; C_7_H_4_N_2_O_7_, 6.3 g L^−1^; NaOH, 20.1 g L^−1^; C_6_H_5_OH, 5 g L^−1^; Na_2_SO_3_, 5 g L^−1^) was added to react in boiling water for 10 min, after which OD was determined at 520 nm. CBH activity assay was carried out as follows: 25 μL PNPC (5 mM), 25 μL NaAc buffer, 30 μL ddH_2_O, and 20 μL crude enzyme solution were mixed, and the system was kept at 50 °C for 20 min, and then 100 μL Na_2_CO_3_ (1 M) was added, after which OD was determined at 402 nm. One enzyme activity unit was defined as the amount of enzyme required to liberate 1 µmol glucose or pNP per minute under assayed conditions [57, 58].

### Intracellular Cys, Met, and GSH content assay

Hyphae were washed several times with deionized water and then rapidly frozen with liquid nitrogen and milled. The homogenate was dissolved in the extraction solution (mass/volume of extracting solution = 1 / 5) and 8000 × *g* centrifuged at 4 °C for 10 min, and the supernatant was kept on ice for subsequent tests. Cysteine/Methionine Assay Kit (Abcam, UK) and Glutathione content detection Kit (Boxbio, China) were used to determine the content of intracellular Cys, Met, and GSH.

### Biomass assay

The hyphae and medium (straw) were fully mixed, and 10.0 g sample was accurately weighed. The samples were rapidly frozen with liquid nitrogen and milled. Hyphae DNA was extracted by the PowerSoil Pro Kit (QIAGEN, Germany) according to the manufacturer's instructions and then dissolved with the same volume of ddH_2_O. Copies of DNA solution: 4.5 × 10^10^ copy μL^−1^ (2000 bp, 100 ng μL^−1^), dilute 10 folds successively, and the Cts of each treatment were determined by qPCR. The standard curve equation is Y = (34.3 − X)/3.2, R^2^ = 0.999, Y = log_10_^(copies of template)^, X = Average of Cts.

### Gene knockout and overexpression in NJAU4742

The homologous arms were obtained through a PCR kit (TaKaRa, Japan). hygromycin phosphotransferase gene (*hph*) and the uridine synthetase gene (*ura*3) were the biomarkers. The functional fragment structure of knockout and overexpression is “upstream-*hph*/*ura*3-downstream” and “upstream-*hph*/*ura3*-promoter-gene” respectively. 100 μL spores (1 × 10^8^ spores mL^−1^) were coated on PDA covered with cellophane, the newly germinated spores were lysed by Solution A (Sorbitol, 6 M; KH_2_PO_4_, 0.5 M; pH was adjusted to 5.6 by KOH) containing fungal cell-wall lyase (SIGMA, US) and protoplasts.

Protoplasts, 400 μL Solution B (Sorbitol, 10 M; CaCl_2_·2H_2_O, 0.5 M; 5 mM pH 7.5 Tris–HCl), 100 μL PEG solution (PEG6000, 25%, W/V; CaCl_2_·2H_2_O, 0.5 M; 5 mM 7.5 Tris–HCl), and 20 μL functional DNA (200 ng μL^−1^) were mixed gently, and then incubated on ice for 20 min, after which 2 mL PEG solution and 3 mL Solution B was gradually added. The mixture was uniformly coated on sucrose PDA (PDA, 39 g L^−1^; sucrose,1 M), cultured at 28 °C for about 18 h, and then covered with Hygromycin PDA (PDA, 39 g L^−1^; Hygromycin B, 0.2 g L^−1^) on coated plate. The transformants were verified by PCR, and the correct transformant need to be verified again after passage [59].

### Western blot

The 6 × His-tag labeled strains were cultured on medium at 28 °C for 3 days, and then total protein was extracted by RIPA lysis buffer (Beyotime, China). The protein was transferred to the nitrocellulose membrane after SDS-PAGE, and then blocked with TBST (Solarbio, China) containing 5.0% non-fat milk. After being incubated with mouse monoclonal 6 × His-tag antibody (Beyotime, China) at 4 °C overnight, the membrane was incubated with Goat-anti-Mouse IgG (H + L) Alexa Fluor 680 labeled antibody (ThermoFisher, US) at normal temperature for 2 h. Finally, the blot was detected by Protein Immunoblotter (Bio-Rad, US).

### RNAi assay

pSilent-1 is a shuttle plasmid with hygromycin and ampicillin resistance gene, and it has two polyclonal sites on both sides of the *Magnaporthe* *grisea* cutinase gene intron [60, 61]. Using the One Step Cloning Kit (Vazyme Biotech, China), the sense chain of target gene was inserted into polyclonal sites, and the other with the anti-sense chain was inserted into polyclonal sites. The vector can express the stem-loop structure RNA that could be recognized and cut by Dicer to form a Silencing-complex (RISC). RISC could specifically identify and cut the RNA transcribed by the target gene [62] (Additional file 1: Fig. S1a). According to the method of transformation mentioned above, the correct vector was transformed and verified by PCR.

### Construction of the glucose FRET sensor functional strain

The FRET sensor gene was cloned from pDR-GW FLII12Pglu-700μ∆6, and the *hph* gene was cloned from pSilent-1, while other DNA fragments were cloned from the NJAU4742 genome. The above cloned gene fragments were fused by fusion PCR into a long DNA fragment with gene expression function, which is structured as “*ura*3-*hph*-*ura*3 upstream-promoter-sensor-*ura*3 downstream”. The fragments were transferred into the protoplasts by using the transformation method mentioned above (PEG/CaCl_2_ transformation method), The ura3 and ura3 downstream act as homology arms and recombine homologously with the NJAU4742 genome. Since the inserted fragment contains ura3 upstream, this results in the presence of repetitive sequences in the inserted fragment and the genome, which induces DNA repair and results in the loss of the fragment between the repetitive sequences. Taking advantage of this feature, we can knock out the ura3 gene while inserting the FRET sensor gene into the genome, and *hph* gene will be lost along, so that we can obtain a ura3-deficient FRET sensor strain for subsequent gene editing on this strain. The *ura*3 gene could express nucleoside lactide-5'-monophosphate decarboxylase, resulting in 5-Fluoroorotic acid being converted to a toxic form (5-fluorouracil). Therefore, 5-Fluoroorotic acid (1 mg mL^−1^) was used to screen the *ura*3 deletion transformant (Additional file 1: Fig. S1C). The target transformants were obtained by PCR laser scanning microscope verification (LSM 980, ZEISS, Germany), Setting up dual-channel simultaneous detection of CFP and YFP fluorescence intensity, YFP and CFP are excited at 500 nm and 430 nm respectively, and emission wavelength of YFP and CFP at 525 nm and 470 nm respectively; The transformant hyphae was incubated in a glucose gradient solution, and the fluorescence intensity of CFP and YFP were detected respectively, and an increase in *F*_YFP_/*F*_CFP_ with gradient indicating the glucose FRET sensor could work successfully in NJAU4742.

### Yeast one-hybrid assay

By using the Ultra One Step Cloning Kit (Vazyme Biotech, China), the promoter region of *Tgmst*1/2 was inserted into the polyclonal site of pAbAi, and the CDS of GRP was inserted into the polyclonal site of pGADT7. Based on PFG/LiAc transformation method, 100μL Gold Yeast, 50 μL NaCl (0.9%, W/V), 5 μL DTT (1 M), 500 μL PEG Mix (45 mL 50% PEG3350; 5 mL 1 M LiAc; 500 μL 1 M Tris–HCl and 100 μL 0.5 M EDTA were mixes) and 1 μg pAbAi-*Tgmst*P linearized by BstBI were mixed gently and incubated at 200 rpm and 30 °C for 30 min, and then 20 μL DMSO was added. The prepared system was heat shocked at 42 °C for 15 min and then kept on ice for 5 min. After resuscitation, the yeast was coated on SD-Ura solid medium (Takara, Japan) and cultured at 30 °C for about 2–3 Days. The transformant (Bait-reporter) was verified by PCR. 10 μL transformant solution (OD_600_ = 0.8) was dropped onto SD-Ura medium containing AbA (400 ng mL^−1^) for self-activation verification. The pGADT7-GRP was transformed into the Bait-reporter and verified by PCR. Similarly, 10 μL transformant solution was dropped onto SD-Ura-Leu medium containing AbA (400 ng mL^−1^), and then cultured at 30 °C for 3 days, until the colony formed.

### Yeast two-hybrid assay

By using the Ultra One Step Cloning Kit (Vazyme Biotech, China), the CDS of *TgpkA* was inserted into the polyclonal site of pGADT7, and the CDS of *Tgmst*1 was inserted into pGBKT7. According to the PFG/LiAc transformation method, pGBKT7-MST1/2 and pGADT7-EV were transformed into Y2H Gold Yeast. The transformants were verified by PCR and sequencing. 10 μL transformant solution (OD_600_ = 0.8) was dropped onto SD-His-Trp solid medium (Takara, Japan), and then cultured at 30 °C for self-activation verification. pGADT7-PKA was transformed into the previous transformant and verified by PCR. 10 μL transformant solution was dropped onto SD-His-Leu-Trp solid medium (Takara, Japan), and then cultured at 30 °C for about 4 days until colony formed.

### ChIP-qPCR

The DNA fragment “*Tggrp* upstream-*hph*-promoter-*Tggrp*-6 × His-*Tggrp* downstream” was transformed and verified by sequencing and Western blot. Hyphae were immersed in Cross-linking buffer (Sucrose, 0.4 M; pH 8.0 Tris–HCl, 10 mM; EDTA, 1.0 mM; FMSF, 1 mM; Formaldehyde, 1%, V/V) for 25 min, and then terminated by 10 × Glycine (Beyotime, China). Subsequently, the cross-linked hyphae were fully milled and the genome was fragmented to 200–1000 bp. 1.8 mL ChIP Diffusion Buffer (Beyotime, China) and 1 μg 6 × His-tag ChIP-class antibody (Abcam, UK) were added into 200 μL supernatant, and incubated at 4 °C overnight. Afterward, 60 μL Protein A/G beads were added into the ChIP system and incubated at 4 °C for 4 to 5 h. The supernatant was removed and the sediment was cleaned by Low Salt Immune Complex Wash buffer, High Salt Immune Complex Wash buffer, LiCl Immune Complex Wash buffer, and TE buffer (Beyotime, China) in turn. Finally, 500 μL Elution Buffer (SDS, 1%, W/V; NaHCO_3_, 0.1 M) was added to elute the Protein-DNA complex on Protein A/G beads and the nucleic acid was collected after de-crosslink.

The collected nucleic acid of IP, Mock (IgG), and Input was diluted to the same concentration. By using TB Green Premix Ex Taq Kit (Takara, Japan), qPCR was performed by multiple primers for the *Tgmst* promoter. Percent Input (% Input) and Fold Enrichment statistical methods were performed. Input is defined as 1.0%., and the Input dilution factor (IDF = 6.644) was subtracted. The formulas were as follows [63]: IDF = log_2_^100^ = 6.644 (1.0% Input); Adjusted input = Raw Ct – IDF; %Input _IP/Mock_ = 100 × 2 ^ (Adjusted Input-Ct _IP/Mock_) × 100%; Fold enrichment = 2 ^ [ (Ct _Mock_) – (Ct _IP_)].

### DNA pull-down assay

The promoter region of *Tgmst* was amplified with biotin-labeled primers, and the product was used as a probe. Nucleoprotein was extracted by CelLytic™ PN Isolation/Extraction Kit (Sigma-Aldrich, US). 1 µg DNA probe and 70 µg nucleoprotein were premixed, and the pull-down system was incubated at 4 °C overnight. Streptavidin magnetic beads were added into the pull-down system, and incubated at 4 °C for 1 h. the protein was pulled down by magnetic frame and used for SDS-PAGE. Differential bands were removed and decolored. DTT (10 mM) and IAM (55 mM) were added in turn for reduction and alkylation; 500 μL acetonitrile was added and vacuum dried for 5 min; 0.01 μg μL^−1^ trypsin was added and ice bath for 30 min; 300 μL extract solution was added and sonicated for 10 min, and eventually the peptide samples (supernatant) was collected. The peptide samples were dissolved in 2% acetonitrile / 0.1% formic acid and analyzed by Triple TOF 5600 plus mass spectrometer coupled with the Eksigent nanoLC system (AB Sciex, USA). The original files were submitted to ProteinPilot (AB Sciex, USA) for protein identification, the Paragon algorithm was used to compare the *T.* *guizhouense* NJAU4742 proteome database (https://bioinfo.njau.edu.cn/tgn4742/index.php).

### Glutathionylation detection assay

The GRP-His labeled strain was cultured on straw medium at 28 °C for 3 Days, then total protein was extracted by RIPA lysis buffer (Beyotime, China) after the hyphae were fully milled. The nickel ion magnetic bead was washed twice with PBS (Beyotime, China) and diluted by PBS to 50% (W/V). 100 μL nickel ion magnetic bead was added to 1 mL protein and incubated at 4 °C for 10 min, then GRP was isolated by magnetic separator. the purified GRP was diluted by PBS (Containing 1 mM PMSF) to 1 μg μL^−1^. The reaction system was heated for 10 min and performed SDS-PAGE. According to the Western blot method, the rabbit anti-GSH antibody (Cloud-clone, China) was used to specifically identify the GSH residues [28].

### Transcriptome analysis

Total RNA was extracted by mirVana miRNA Isolation Kit (Ambion, US). RNA integrity was evaluated by Agilent 2100 Bioanalyzer (Agilent, USA). The samples with RNA Integrity Number (RIN) ≥ 7 were subjected to subsequent analysis. The libraries were constructed by using mRNA LTSample Prep Kit (Illumina, USA). Then these libraries were sequenced by the Illumina sequencing platform and 125 bp / 150 bp paired-end reads were generated. FPKM value of each gene was calculated, and the read counts of each gene were obtained by htseq-count. DEGs were identified by using DESeq (2012) R package functions estimateSizeFactors and nbinomTest. *P* value < 0.05 and FoldChange > 2 or FoldChange < 0.5 were set as thresholds for significantly differential expression [64]. Hierarchical cluster analysis of DEGs was performed to explore gene expression patterns [65]. GO enrichment and KEGG pathway enrichment analysis of DEGs was performed by R based on hypergeometric distribution. The alternatively splicing analysis of differentially regulated transcripts, isoforms, or exons was performed by ASprofile. SNP and INDEL were called by using samtools and bcftools, then the effects of variants on genes were annotated and predicted by snpeff [66].

### Metabolomics analysis

Fresh WT spores were incubated in T1 and T3 treatments mentioned above at 28 °C for 5 days, and each treatment was set up with 6 biological repeats. The hyphae were fully milled and dissolved by extraction buffer (Methanol/acetonitrile/water = 2/2/1, V/V), and centrifuged at 10,000 × g for 2 min. The supernatant was filtered through 0.25 µm filters. The substances in samples were analyzed by liquid chromatography-mass spectrometry (LC–MS/MS). The raw data were logarithmically transformed and centered by using SIMCA (V16.0.2, Umea, Sweden) and then analyzed by automatic modeling. Subsequently, UV formatting and OPLS-DA modeling analysis were performed for principal component analysis. The adjusted data were compared with the metabolite database and combined with univariate statistical analysis (UVA) and multivariate statistical analysis (MVA). Finally, a series of bioinformatics analyses were performed to visualize the biological functions of differential metabolites.

### Supplementary Information

**Additional file 1: Fig. S1.** Cys determination and glutathionylation detection of GRP. (A) Intracellular Cys content in different sulfate-content treatments, wild-type NJAU4742 was grown in the treatments at 28 °C for 5 days. (B) The strain GRP-His was incubated under high sulfur conditions, and glutathionylation of GRP was detected. Rabbit anti-GSH antibody was used as the primary antibody and the hybridization signal could be detected. **Fig. S2.** The schematic diagram of the construction principle of RNAi, FRET sensor, and *ura*3 deficient strains. (A) The vector construction method and working principle of RNA interference of *Tgatps.* The green and blue fragments respectively express Sense and Antisense chain, and the two fragments are linked by *Magnaporthe* *grisea* cutinase gene intron (yellow), TrpC Promoter (TrpC P) and TrpC terminator (TrpC T) to start and stop the transcription of the functional fragment, and Hygromycin resistance gene (*hph*) was used as the biomarker. It could eventually express a stem-loop RNA, which would be recognized and cut by Dicer, and form an RNA interference silencing complex (RISC). (B) Diagram of structure and working principle of the Optical intracellular glucose FRET sensor. The glucose-binding protein subunit MglB connects CFP (blue) and YFP (yellow). The color and length of the wavy line (red is excitation light) represented the fluorescent category and intensity, respectively. Glucose binding MglB would change the relative spatial position of CFP and YFP, then lead to the change of FRET, and finally cause the change of *F*_Y_/*F*_C_. (C) Diagram of the *ura*3 deficiency strain construction principle that expressed the glucose FRET sensor. Make the target DNA fragment (the top one) replace *ura3* and *ura3* downstream (2kb) through homologous arm recognition. It made two repetitive sequences appear in the near region of the genome, which would cause the DNA repair mechanism to cause DNA (*ura3* and *hph*) loss between repetitive sequences. 5-FOA itself was non-toxic to NJAU4742 cells, *ura*3 could express nucleoside lactide-5’-monophosphate decarboxylase, which would be transformed into a toxic form (5-fluorouracil) so that cells couldn’t grow. 5-FOA (1 mg mL^-1^) was used to screen correct transformants. **Fig. S3.** The IPR statistic, KEGG, and GO enrichment of pull-down products. (A) Schematic diagram of the biotin-primer amplification region, the bait sequence was amplified from the -2000bp to -1bp region of *Tgmst*. (B) Results of structural domain annotation of all identified proteins in the EXP group; among them, a higher percentage of Hsp70 protein domains were detected. (C) KEGG Pathway annotation results for all identified proteins in the EXP group. (D) Gene ontology annotation results for all identified proteins in the EXP group. **Fig. S4.** The Protein-DNA interaction between GRP and *Tgmst*1 promoter was verified by ChIP-qPCR. (A) Western blot of GRP labeled by His-tag was added, and three repeats were used to verify the successful addition of His-tag. (B) qPCR primers for ChIP products. (C) Statistical process of ChIP-qPCR; (C1) The specific recognition site of these primers was within 1200 bp upstream of the *Tgmst*1 promoter, and their amplification products are about 200bp and different from each other. The Cts detected by P-5 and P-6 were significantly different between IP and Mock, and the most significant difference was detected by P-6, which amplified the region from -363 bp to -224 bp. (C2) The percentage of *Tgmst*1 promoter fragments in the immunoprecipitated DNA of IP and Mock was calculated. input (1%) was adjusted to 100% and its Ct was 16, and the *Tgmst*1 promoter region in IP accounted for 17.6% of the precipitated DNA fragments by equation 2^ (16 - Ct), while it was 0.296% in Mock (IgG), indicating that GRP has significant DNA-protein interactions with the *Tgmst* promoter region. (C3) The precipitation efficiency of IP on the *Tgmst*1 promoter fragment was 61.6 folds higher than that of Mock (IgG), indicating a strong interaction between GRP and the *Tgmst*1 promoter region, with the interaction region ranging from -363 bp to -224 bp. **Fig. S5.** EG, CBH, and Xylanase activity of different treatments and strains. (A1, 2, 3) The EG, CBH, and Xylanase activities of NJAU4742 in different sulfate-content MM+straw, grown at 28 °C for 5 days. (B1, 2, 3) The EG, CBH, and Xylanase activities of WT, OE-*Tgatps*, ∆*Tgatps*, and RNAi-*Tgatps* on MM+straw, strains were grown at 28 °C for 5 days. (C1, 2, 3) The EG, CBH, and Xylanase activities of WT, OE-*TgcysK*, ∆*TgcysK*, OE-*Tghmt*, and ∆*TgcysK* on MM+straw, strains were grown at 28 °C for 5 days. (D1, 2, 3) The EG, CBH, and Xylanase activities of WT, OE-*Tgmst*, and ∆*Tgmst*, on MM+straw, strains were grown at 28 °C for 5 days. (E1, 2, 3) The EG, CBH, and Xylanase activities of WT, OE-*Tggrp*, ∆*Tggrp* on MM+straw, strains were grown for 5 days at 28 °C. Bars represent mean ± SEM, with n = 3 biologically independent experiments; red dots resemble values from individual experiments. ANOVA was conducted in (A), and there was a significant effect of inorganic sulfide on the EG, CBH, and Xylanase activities at the *P*< 0.05 level for the 4 treatments, post hoc comparisons using the Tukey's HSD test indicated that the mean score for the EG, CBH, Xylanase activities of T2 were significantly greater than that of T1; and T1 was significantly greater than T3. However, T4 did not significantly differ from T3. Student’s *t*-testing was conducted in (B, C, D, E), *significant difference to WT at two-tailed *P* = 0.017 (B1, OE-*Tgatps*), 0.031 (B3, OE-*Tgatps*); **significant difference to WT at two-tailed *P* = 0.0018 (B1, RNAi-*Tgatps*), 0.0080 (B2, OE-*Tgatps*) ***significant difference to WT at two-tailed *P* = 0.0000020 (B1, ∆*Tgatps*), 0.00079 (B2, ∆*Tgatps*), 0.00054 (B2, RNAi-*Tgatps*), 0.000014 (B3, ∆*Tgatps*), 0.00073 (B3, RNAi-*Tgatps*); *significant difference to WT at two-tailed *P* = 0.034 (C2, OE-*Tghmt*); **significant difference to WT at two-tailed *P* = 0.0018 (C1, OE-*Tghmt*), 0.0052 (C3, OE-*TgcysK*), 0.0054 (C3, ∆*TgcysK*); ***significant difference to WT at two-tailed *P* = 0.00029 (C1, OE-*TgcysK*), 0.0000060 (C1, ∆*TgcysK*), 0.000042 (C2, OE-*TgcysK*), 0.000017 (C2, ∆*TgcysK*); no significant difference to WT at two-tailed *P* = 0.480633 (C1, ∆*Tghmt*), 0.61 (C2, ∆*Tghmt*), 0.15 (C3, OE-*Tghmt*), 0.60 (C3, ∆*Tghmt*); **significant difference to WT at two-tailed *P* = 0.0025 (D1, OE-*Tgmst*), 0.0028 (D1, ∆*Tgmst*), 0.0084 (D2, OE-*Tgmst*), 0.0024 (D2, ∆*Tgmst*), 0.0076 (D3, OE-*Tgmst*); no significant difference to WT at two-tailed *P* = 0.070 (D3, ∆*Tgmst*); *significant difference to WT at two-tailed *P* = 0.022 (E1, OE-*Tggrp*), 0.018 (E1, ∆*Tggrp*), 0.030 (E2, OE-*Tggrp*), 0.012 (E3, OE-*Tggrp*); **significant difference to WT at two-tailed *P* = 0.0049 (E2, ∆*Tggrp*), 0.0055 (E3, ∆*Tggrp*). **Fig. S6.** Original images of Y1H, Y2H, and Western blot analysis results. (A) Original image of yeast one-hybrid; the growth on SD-Ura-Leu + AbA medium of the yeast transformants that pABAi-*Tgmst*1P and pGADT7-ATPeF1B / pGADT7-GRP / pGADT7-KatG / pGADT7-ATPeF1A / pGADT7-EF5A / pGADT7-EV were co-transformed. (B) Original image of yeast one-hybrid; the growth on SD-Ura-Leu + AbA medium of the yeast transformants that pABAi-*Tgmst*P and pGADT7-GRP / pGADT7-EV were co-transformed. (C) Original images of yeast two-hybrid, (c1) The growth on SD-His-Leu-Trp medium of yeast transformant with pGADT7-PKA and pGBKT7-MST1 were co-transformed; (c2) The growth on SD-Ade-His-Leu-Trp medium of yeast transformant with pGADT7-PKA and pGBKT7-MST1 were co-transformed; (c3) The growth on SD-His-Leu-Trp medium of yeast transformant with pGADT7-EV and pGBKT7-MST1 were co-transformed. (D) Original images of Western blot; first incubated with mouse anti-His-tag primary antibody, washed with TBST, and then incubated with mouse primary β-actin antibody, and finally incubated with secondary antibody. FoldChange of the expression levels of MST1 and MST2 in OE-*Tggrp* relative to WT, with β-actin as an internal reference. (E) Original images of Western blot; effect of different glutathionylation levels of GRP on the expression level of MST1 and MST2, with β -actin as an internal reference. **Fig. S7.** The metabolic pathway of NJAU4742 affected by exogenous inorganic sulfide addition. The area of the square indicates the size of the influence factor of the pathway in the topological analysis, the larger the size the larger the influence factor; the color of the square indicates the -ln *P*-value of the enrichment analysis, and the darker the color the smaller the *P*-value. The treemap results displayed the mainly changed metabolic pathways in T2 relative to T1, Glutathione metabolism and Cysteine & methionine metabolism were significantly affected under the conditions of exogenous inorganic sulfide addition, which was in accordance with our previous assay, suggesting that increased the addition of inorganic sulfide favored cysteine and glutathione synthesis. In addition, mainly some amino acid synthesis pathways, such as alanine, aspartate, glutamate, valine, leucine, etc., responded to the increase of exogenous inorganic sulfide content. The pyruvate metabolism, a key precursor for cysteine production, was likewise significantly affected under this condition. **Fig. S8.** LC-MS/MS and database comparison showed the intracellular metabolites of NJAU4742 affected by exogenous inorganic sulfide addition.

**Additional file 2.** Table introduction.

**Additional file 3.** The differentially expressed gene of OE-*TgcysK* and ∆*TgcysK* relative to WT.

**Additional file 4.** The differentially expressed gene of OE-*Tghmt* and ∆*Tghmt* relative to WT relative to WT.

**Additional file 5.** The Pull-down protein identified in the control (CON) group.

**Additional file 6.** The Pull-down protein identified in the experiment (EXP) group.

**Additional file 7.** The mass spectrometry identification result of intracellular substances of hyphae grown under T1 and T3 treatment conditions.

## Data Availability

The referenced data can be obtained from the corresponding article and NCBI. Some of the original data in this study are represented in the supplementary material, and some data are being published.
