# Supplementary material for: Cysteine facilitates the lignocellulolytic response of Trichoderma guizhouense NJAU4742 by indirectly up-regulating membrane sugar transporters
Source: Biotechnol Biofuels Bioprod. 2023 Oct 27;16:159. doi: 10.1186/s13068-023-02418-9 (PMC10612256; doi:10.1186/s13068-023-02418-9)
Supplement: Supplementary file 1 — Additional file 1: Fig. S1. Cys determination and glutathionylation detection of GRP. (A) Intracellular Cys content in different sulfate-content treatments, wild-type NJAU4742 was grown in the treatments at 28 °C for 5 days. (B) The strain GRP-His was incubated under high sulfur conditions, and glutathionylation of GRP was detected. Rabbit anti-GSH antibody was used as the primary antibody and the hybridization signal could be detected. Fig. S2. The schematic diagram of the construction principle of RNAi, FRET sensor, and ura3 deficient strains. (A) The vector construction method and working principle of RNA interference of Tgatps. The green and blue fragments respectively express Sense and Antisense chain, and the two fragments are linked by Magnaporthe grisea cutinase gene intron (yellow), TrpC Promoter (TrpC P) and TrpC terminator (TrpC T) to start and stop the transcription of the functional fragment, and Hygromycin resistance gene (hph) was used as the biomarker. It could eventually express a stem-loop RNA, which would be recognized and cut by Dicer, and form an RNA interference silencing complex (RISC). (B) Diagram of structure and working principle of the Optical intracellular glucose FRET sensor. The glucose-binding protein subunit MglB connects CFP (blue) and YFP (yellow). The color and length of the wavy line (red is excitation light) represented the fluorescent category and intensity, respectively. Glucose binding MglB would change the relative spatial position of CFP and YFP, then lead to the change of FRET, and finally cause the change of FY/FC. (C) Diagram of the ura3 deficiency strain construction principle that expressed the glucose FRET sensor. Make the target DNA fragment (the top one) replace ura3 and ura3 downstream (2kb) through homologous arm recognition. It made two repetitive sequences appear in the near region of the genome, which would cause the DNA repair mechanism to cause DNA (ura3 and hph) loss between repetitive sequences. 5-FOA itself [file 13068_2023_2418_MOESM1_ESM.docx]

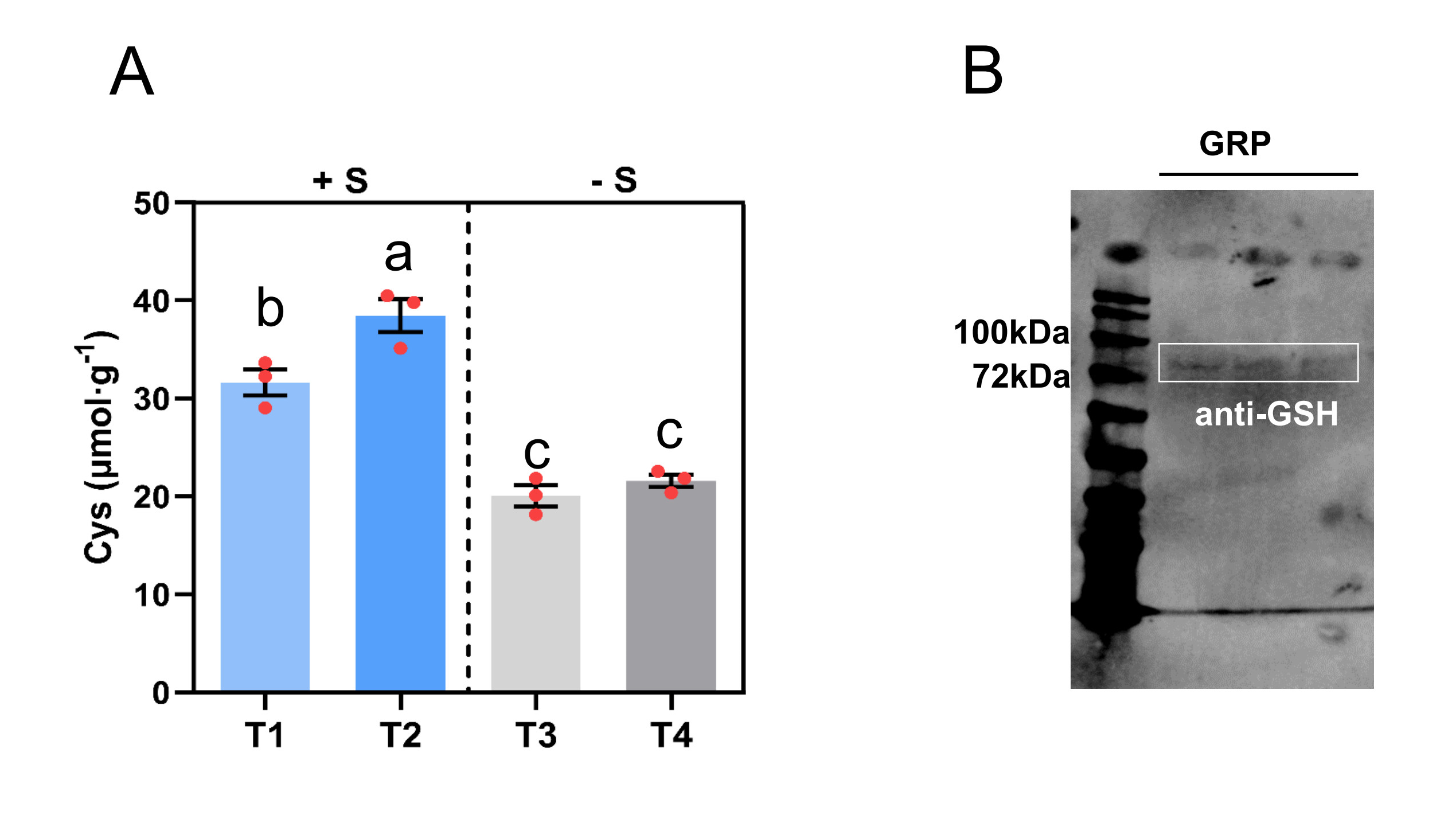


**Fig. S1 Intracellular Cys determination and glutathionylation detection of GRP. (A)** Intracellular Cys content in different sulfate-content treatments, hyphae was grown at 28 °C for 5 days. **(B**)The total proteins of strain GRP-His were extracted, and GRP was purified. in T1, Glutathionylation of GRP was detected by using rabbit anti-GSH antibody as the primary antibody. blot of GRP in the white box was detected by anti-GSH antibody. ANOVA was conducted in (A), Tukey's HSD test was used for post hoc comparisons, and the letters “a”, “b”, and “c” were used for significance exhibition.


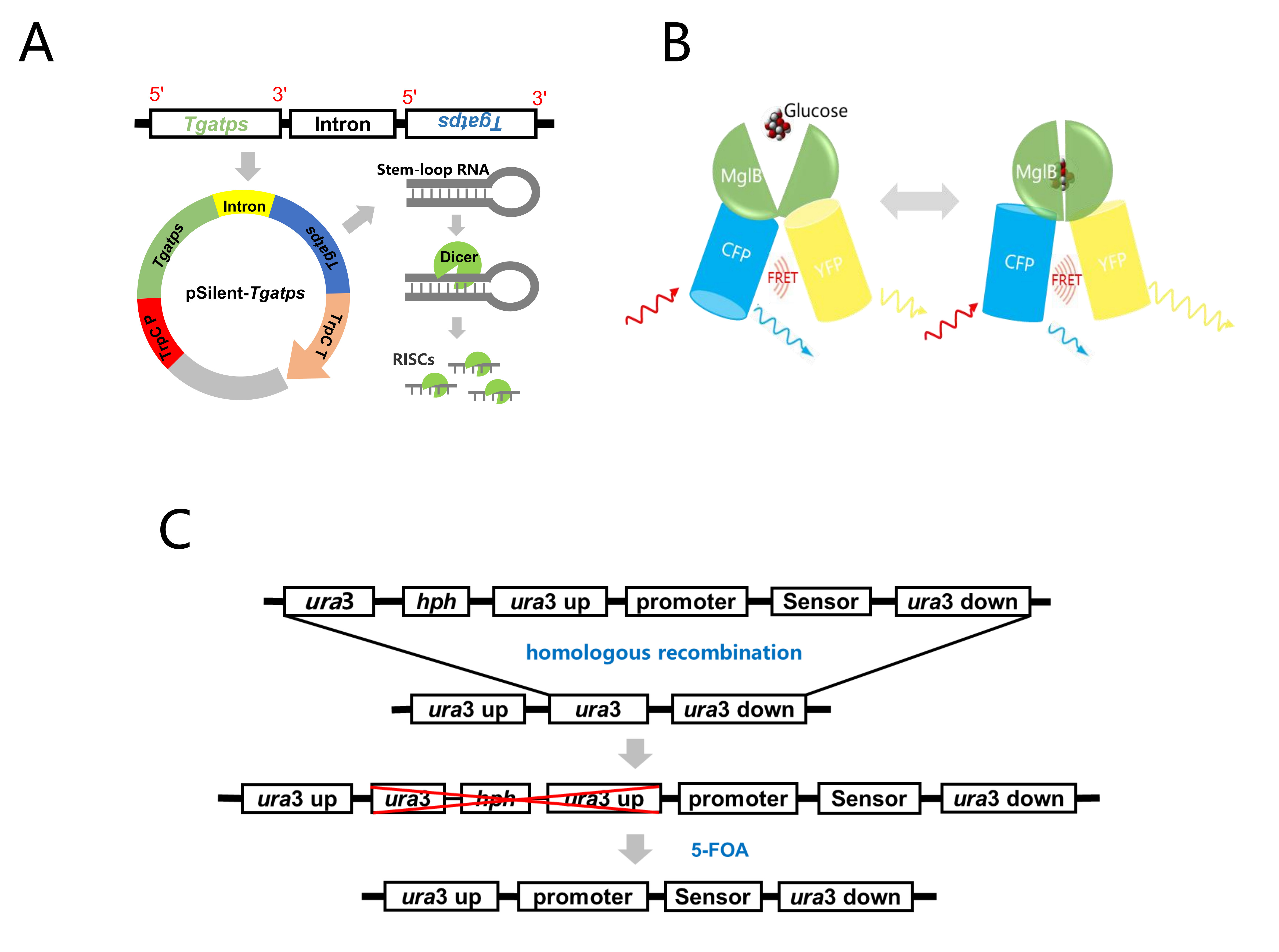


**Fig. S2 The schematic diagram of RNAi, FRET sensor, and** ***ura*3 deficient strain construction. (A)** The green and blue fragment respectively expressed sense and antisense chain, and the two fragments were linked by *Magnaporthe grisea* cutinase gene intron (yellow), TrpC promoter (TrpC P) and TrpC terminator (TrpC T) to start and stop transcription, and Hygromycin resistance gene (*hph*) was used as a biomarker. It could eventually express a stem-loop RNA, which would be recognized and cut by Dicer, and form an RNA interference silencing complex (RISC). **(B)** Structure and working principle of the Optical intracellular glucose FRET sensor. The glucose-binding protein subunit MglB connected CFP (blue) and YFP (yellow). The color and length of wavy line (red was excitation light) represented the fluorescence category and intensity. MglB bound by glucose would change the relative spatial position of CFP and YFP, then lead to the change of FRET and *F*_Y_/*F*_C_. **(C)** Diagram of *ura*3 deficiency strain construction principle. The DNA fragment (the top one) was homologously recombined into genome. The DNA (*ura*3 and *hph*) between repeat sequences was removed from genome by the spontaneous DNA repair mechanism. 5-FOA could be transformed into the toxic form (5-fluorouracil) by *ura*3 expressed nucleoside lactide-5'-monophosphate decarboxylase. Therefore, 5-FOA (1 mg·mL^-1^) was used to screen the ura3 deficient strain.


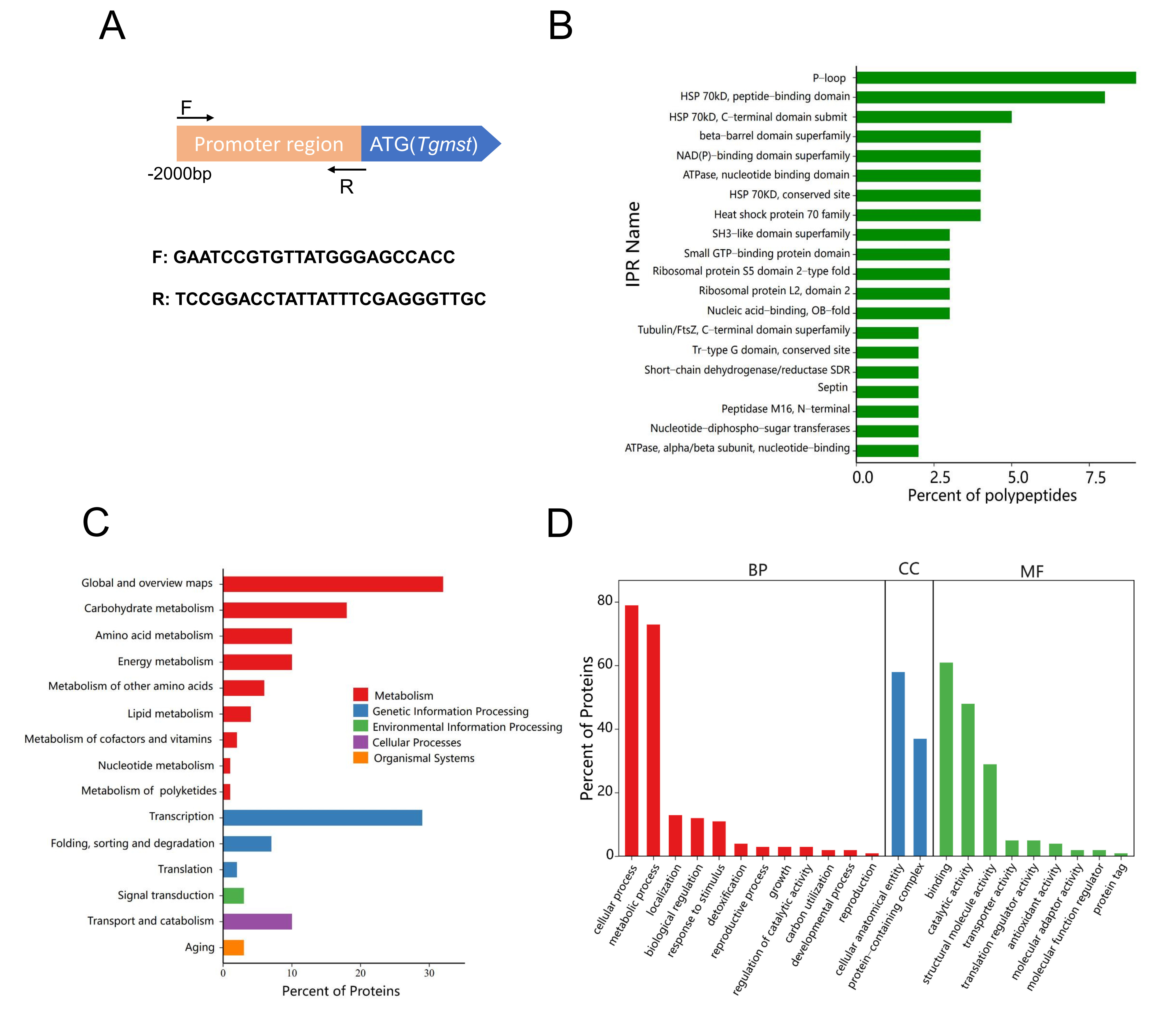


**Fig. S3 The IPR statistic, KEGG, and GO enrichment of pull-down products. (A)** The biotin-primer amplification region, bait was amplified from -2000bp to -1bp of *Tgmst* promoter. **(B)** Structural domain annotation of all identified proteins in EXP group; among them, a higher percentage of Hsp70 protein domain were detected. **(C)** KEGG Pathway annotation for all identified proteins in the EXP group. **(D)** Gene ontology annotation for all identified proteins in the EXP group.


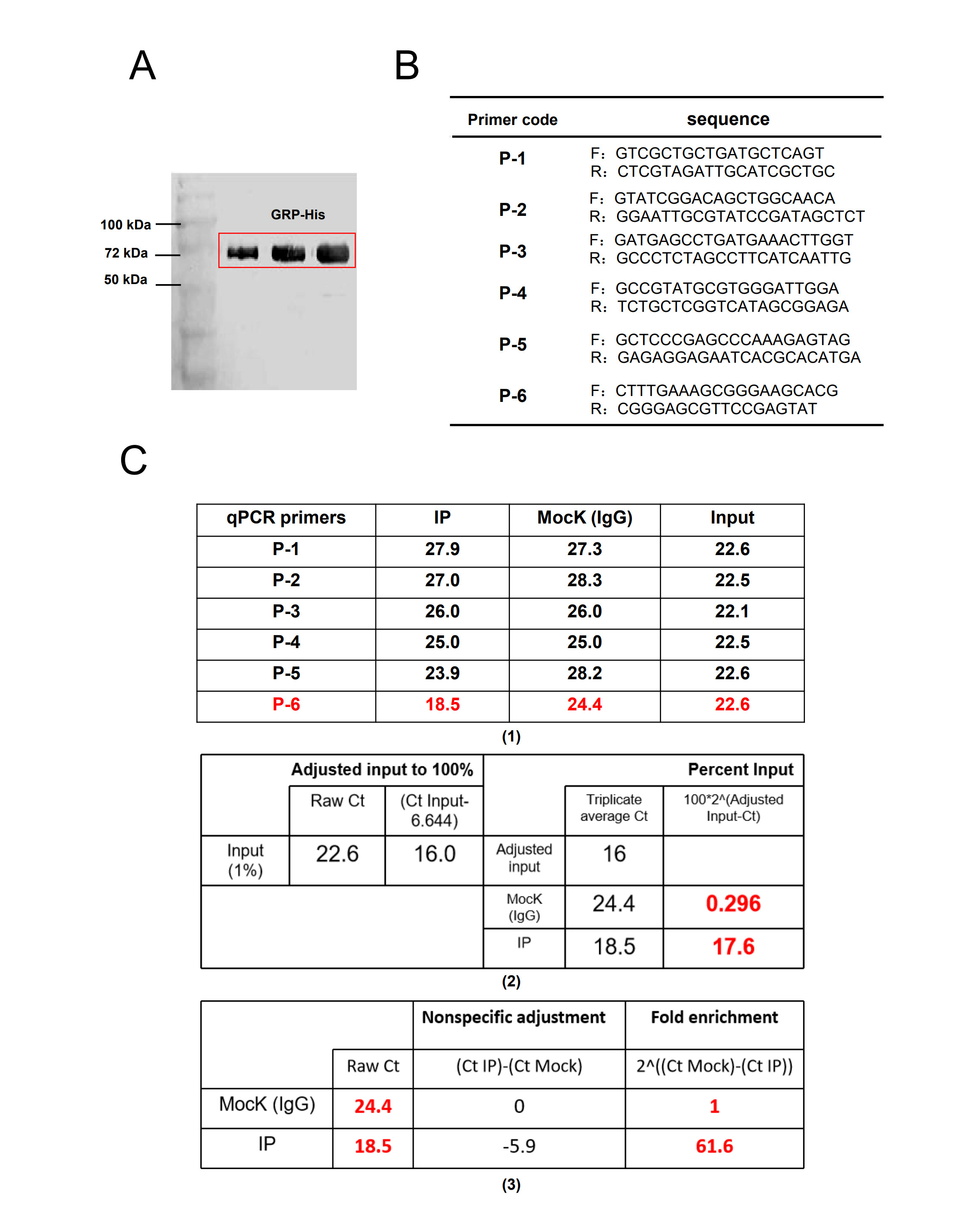


**Fig. S4 The Protein-DNA interaction between GRP and *Tgmst*1 promoter was verified by ChIP-qPCR. (A)** Western blot of GRP verified the successful addition of His-tag. **(B)** qPCR primers sequence for ChIP products. **(C)** Statistical process of ChIP-qPCR; (C1) The specific recognition site of these primers was within 1200 bp of *Tgmst*1 promoter, and their amplification products were about 200bp. The Cts detected by P-5 and P-6 were significantly different in IP and Mock, and the most significant difference was detected by P-6, which amplified the region from -363 bp to -224 bp. (C2) The percentage of *Tgmst*1 promoter fragments in immunoprecipitated DNA of IP and Mock was calculated. The *Tgmst*1 promoter region in IP accounted for 17.6% in precipitated DNA, while it was 0.296% in Mock (IgG) (C3) The precipitation efficiency of IP on *Tgmst*1 promoter fragment was 61.6 folds higher than Mock (IgG).


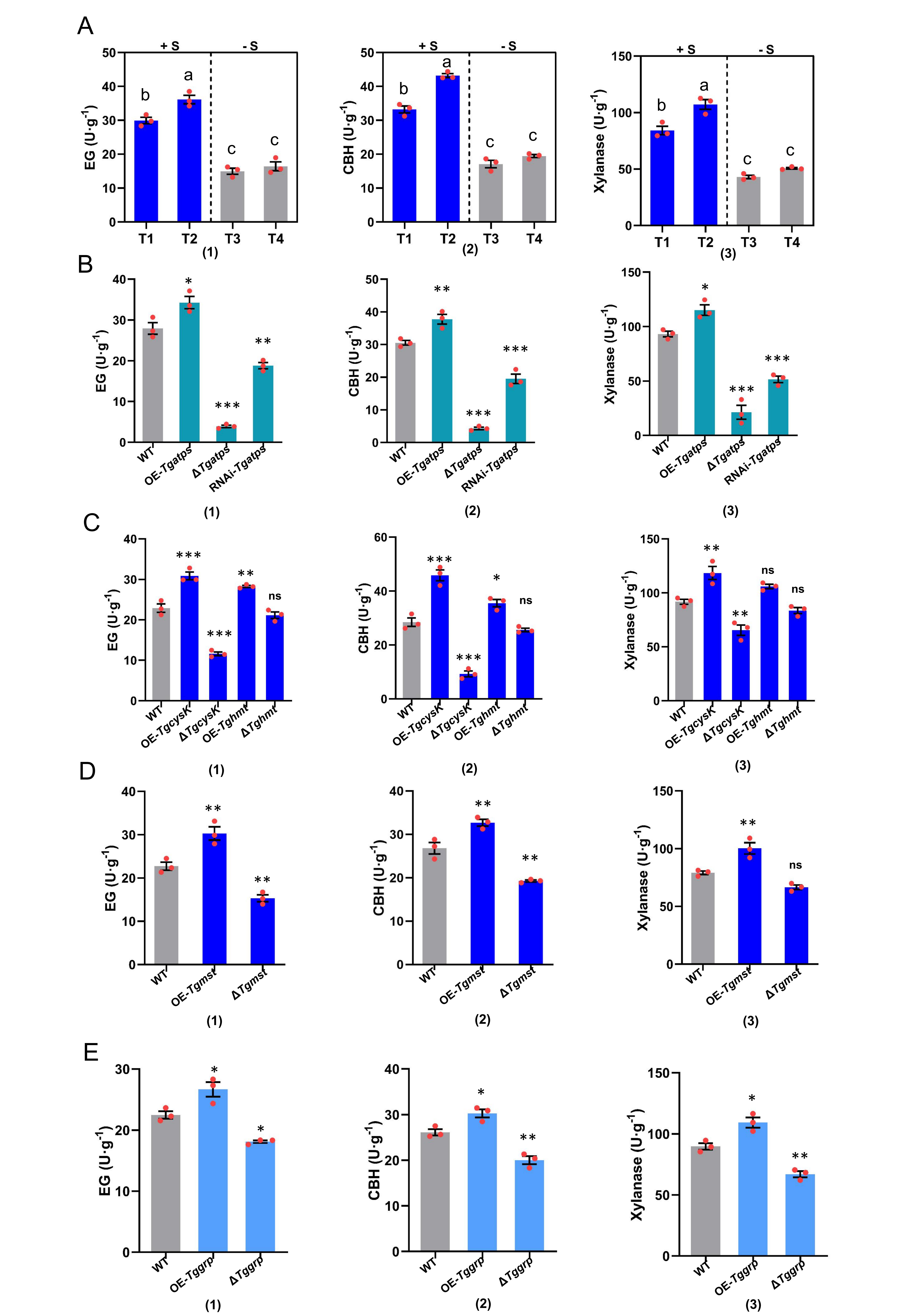


**Fig. S5 EG, CBH, and Xylanase activities of treatments and strains.** **(A1, 2, 3)** The EG, CBH, and Xylanase activities of NJAU4742 in different sulfate-content MM+straw, grown at 28 °C for 5 days. **(B1, 2, 3)** The EG, CBH, and Xylanase activities of WT, OE-*Tgatps*, Δ*Tgatps*, and RNAi-*Tgatps* on MM+straw, strains were grown at 28 °C for 5 days. **(C1, 2, 3)** The EG, CBH, and Xylanase activities of WT, OE-*TgcysK*, Δ*TgcysK*, OE-*Tghmt*, and Δ*TgcysK* on MM+straw, strains were grown at 28 °C for 5 days. **(D1, 2, 3)** The EG, CBH, and Xylanase activities of WT, OE-*Tgmst*, and Δ*Tgmst*, on MM+straw, strains were grown at 28 °C for 5 days. **(E1, 2, 3)** The EG, CBH, and Xylanase activities of WT, OE-*Tggrp*, Δ*Tggrp* on MM+straw, strains were grown for 5 days at 28 °C. Bars represent mean ± SEM, with n = 3 biologically independent experiments; red dots resemble values from individual experiments. ANOVA was conducted in (B, C), Tukey's HSD test was used for post hoc comparisons, and the letters “a”, “b”, and “c” were used for significance exhibition., and there was a significant effect of inorganic sulfide on the EG, CBH, and Xylanase activities (*P* < 0.05). The EG, CBH, and Xylanase activities of T2 were significantly greater than that of T1; and T1 was significantly greater than T3. However, T4 did not significantly differ from T3. Student’s *t*-testing was conducted in (B, C, D, E), *signiﬁcant difference to WT at two-tailed *P* = 0.017 (B1, OE-*Tgatps*), 0.031 (B3, OE-*Tgatps*); **signiﬁcant difference to WT at two-tailed *P* = 0.0018 (B1, RNAi-*Tgatps*), 0.0080 (B2, OE-*Tgatps*) ***signiﬁcant difference to WT at two-tailed *P* = 0.0000020 (B1, Δ*Tgatps*), 0.00079 (B2, Δ*Tgatps*), 0.00054 (B2, RNAi-*Tgatps*), 0.000014 (B3, Δ*Tgatps*), 0.00073 (B3, RNAi-*Tgatps*); *signiﬁcant difference to WT at two-tailed *P* = 0.034 (C2, OE-*Tghmt*); **signiﬁcant difference to WT at two-tailed *P* = 0.0018 (C1, OE-*Tghmt*), 0.0052 (C3, OE-*TgcysK*), 0.0054 (C3, Δ*TgcysK*); ***signiﬁcant difference to WT at two-tailed *P* = 0.00029 (C1, OE-*TgcysK*), 0.0000060 (C1, Δ*TgcysK*), 0.000042 (C2, OE-*TgcysK*), 0.000017 (C2, Δ*TgcysK*); no signiﬁcant difference to WT at two-tailed *P* = 0.480633 (C1, Δ*Tghmt*), 0.61 (C2, Δ*Tghmt*), 0.15 (C3, OE-*Tghmt*), 0.60 (C3, Δ*Tghmt*); **signiﬁcant difference to WT at two-tailed *P* = 0.0025 (D1, OE-*Tgmst*), 0.0028 (D1, Δ*Tgmst*), 0.0084 (D2, OE-*Tgmst*), 0.0024 (D2, Δ*Tgmst*), 0.0076 (D3, OE-*Tgmst*); no signiﬁcant difference to WT at two-tailed *P* = 0.070 (D3, Δ*Tgmst*); *signiﬁcant difference to WT at two-tailed *P* = 0.022 (E1, OE-*Tggrp*), 0.018 (E1, Δ*Tggrp*), 0.030 (E2, OE-*Tggrp*), 0.012 (E3, OE-*Tggrp*); **signiﬁcant difference to WT at two-tailed *P* = 0.0049 (E2, Δ*Tggrp*), 0.0055 (E3, Δ*Tggrp*).


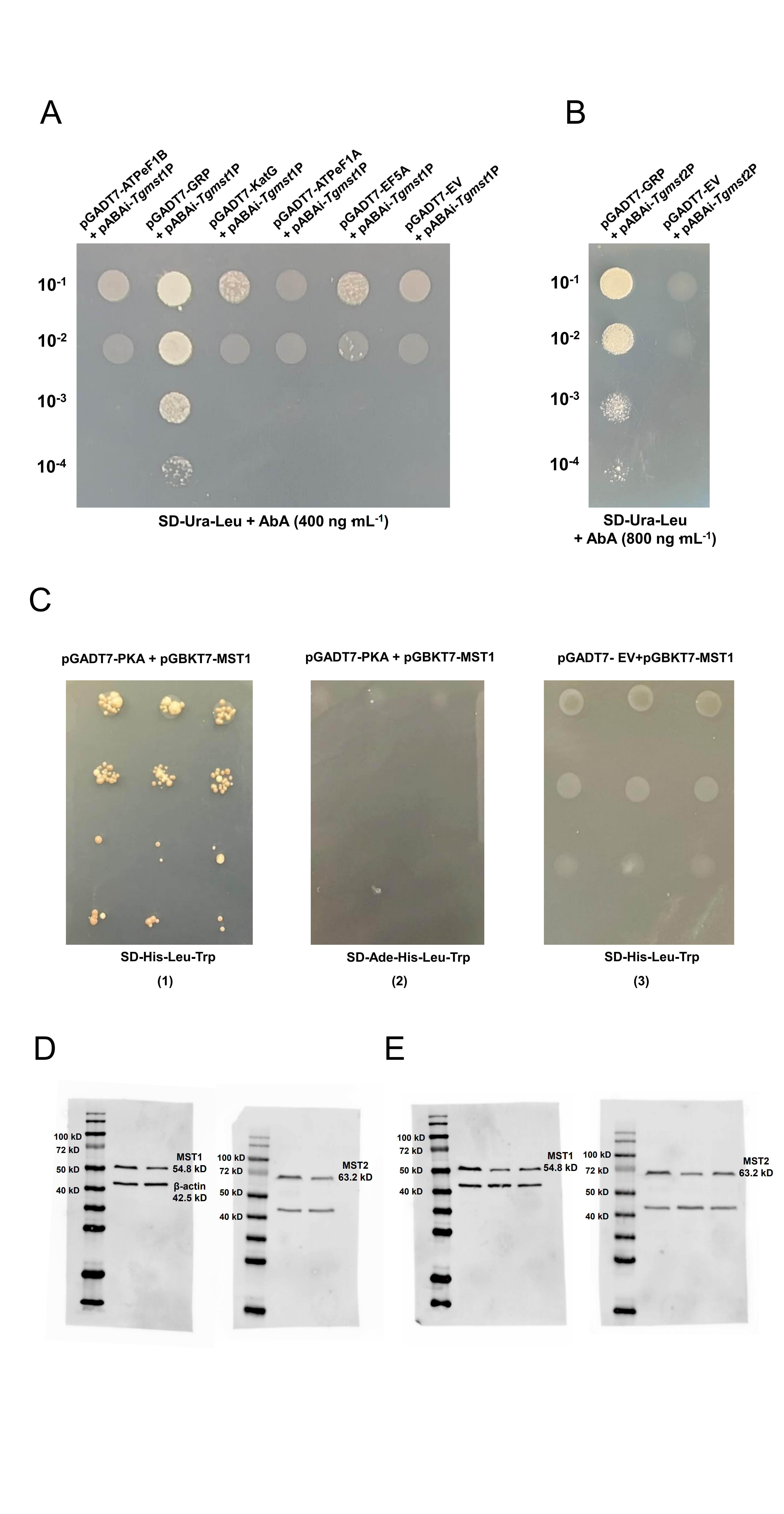


**Fig. S6 Original images of Y1H, Y2H, and Western blot. (A)** Original image of yeast one-hybrid; the growth on SD-Ura-Leu + AbA medium of the yeast transformants that pABAi-*Tgmst*1P and pGADT7-ATPeF1B / pGADT7-GRP / pGADT7-KatG / pGADT7-ATPeF1A / pGADT7-EF5A / pGADT7-EV were co-transformed. **(B)** Original image of yeast one-hybrid; the growth on SD-Ura-Leu + AbA medium of the yeast transformants that pABAi-*Tgmst*P and pGADT7-GRP / pGADT7-EV were co-transformed. **(C)** Original images of yeast two-hybrid. (C1) The growth on SD-His-Leu-Trp medium of yeast transformant with pGADT7-PKA and pGBKT7-MST1 were co-transformed; (C2) the growth on SD-Ade-His-Leu-Trp medium of yeast transformant with pGADT7-PKA and pGBKT7-MST1 were co-transformed; (c3) the growth on SD-His-Leu-Trp medium of yeast transformant with pGADT7-EV and pGBKT7-MST1 were co-transformed. **(D)** Original images of Western blot; the protein was firstly incubated with mouse anti-His-tag primary antibody, washed with TBST, and then incubated with mouse primary β-actin antibody, and finally incubated with secondary antibody. FoldChange of expression levels of MST1 and MST2 in OE-*Tggrp* relative to WT, with β-actin as an internal reference. **(E)** Original images of Western blot; the effect of different glutathionylation levels of GRP on expression level of MST1 and MST2, with β-actin as an internal reference.


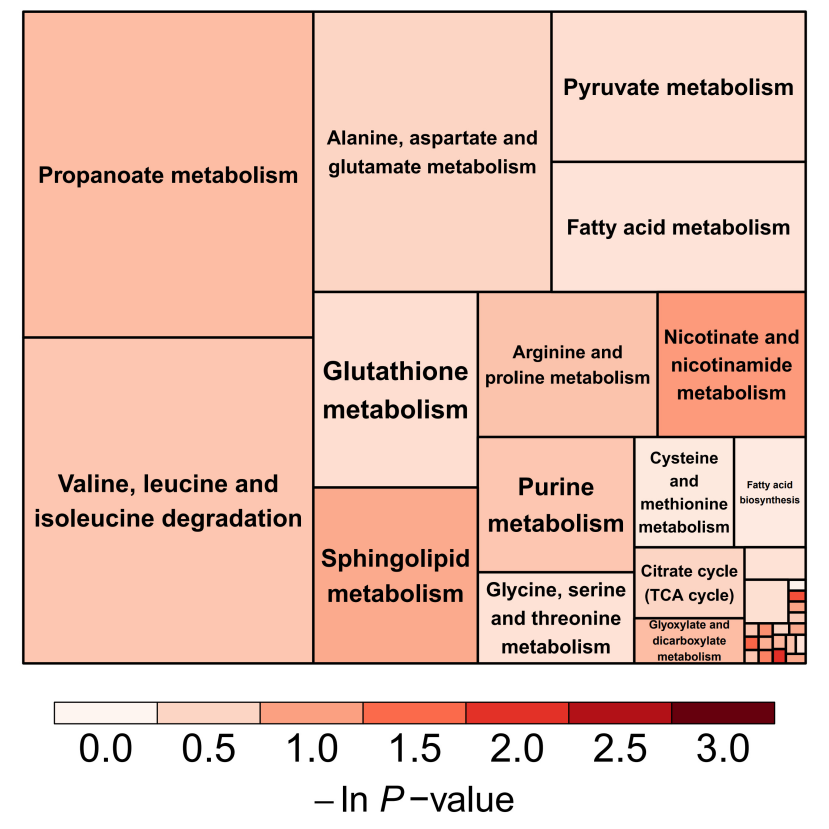


**Fig. S7 The major metabolic pathways in response to inorganic sulfide addition.** The square area indicated the influence factor of pathways in topological analysis, the larger size the larger influence factor; the square color indicated -ln *P*-value of enrichment analysis, and the darker color the smaller *P*-value. The treemap displayed all the mainly changed metabolic pathways in T1 relative to T3. Sulfur addition has broadest effect on various amino acid metabolism, such as alanine, aspartate, cysteine, methionine arginine, proline, valine, leucine, etc. Cys and Met metabolism was identified as a contributor to the lignocellulolytic response among all differential metabolic. pathways. In addition, pyruvate and propanoate metabolism are closely related to the synthesis of Ala, and Ala was the precursor of Cys. The up-regulation of the Cys synthesis pathway induced upregulation of its downstream metabolite GSH, and the screening of Cys metabolism and GSH metabolism by Treemap as the significant differential metabolism was also consistent with the changes in intracellular Cys and GSH that we determined. Interestingly, the TCA cycle was up-regulated, suggesting a more vigorous intracellular energy metabolism, which may be induced by higher intracellular glucose levels. Sphingolipid and fatty acid metabolism were the pathways related to cellular energy storage and were also up-regulated in response to the presence of high intracellular glucose levels.


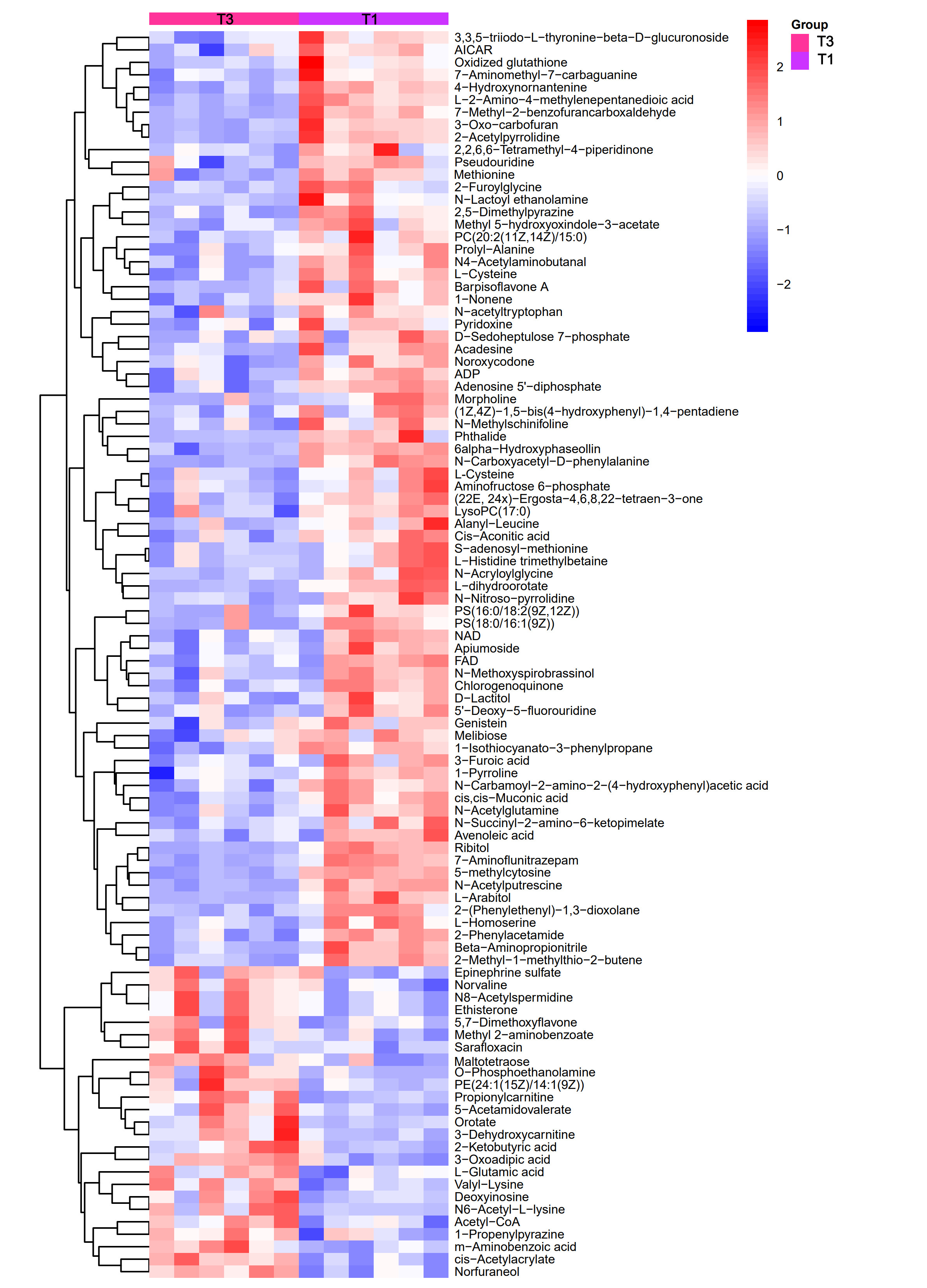


**Fig. S8 Differential metabolites of T1 relative to T3 identified by secondary mass spectrometry.**  The heatmap demonstrated significant differential metabolites with high confidence and low *P*-values. This result was obtained from mass spectrometry signal intensity statistics, which demonstrated content changes of the major intracellular differential metabolites, with cysteine and glutathione identified as significantly up-regulated.


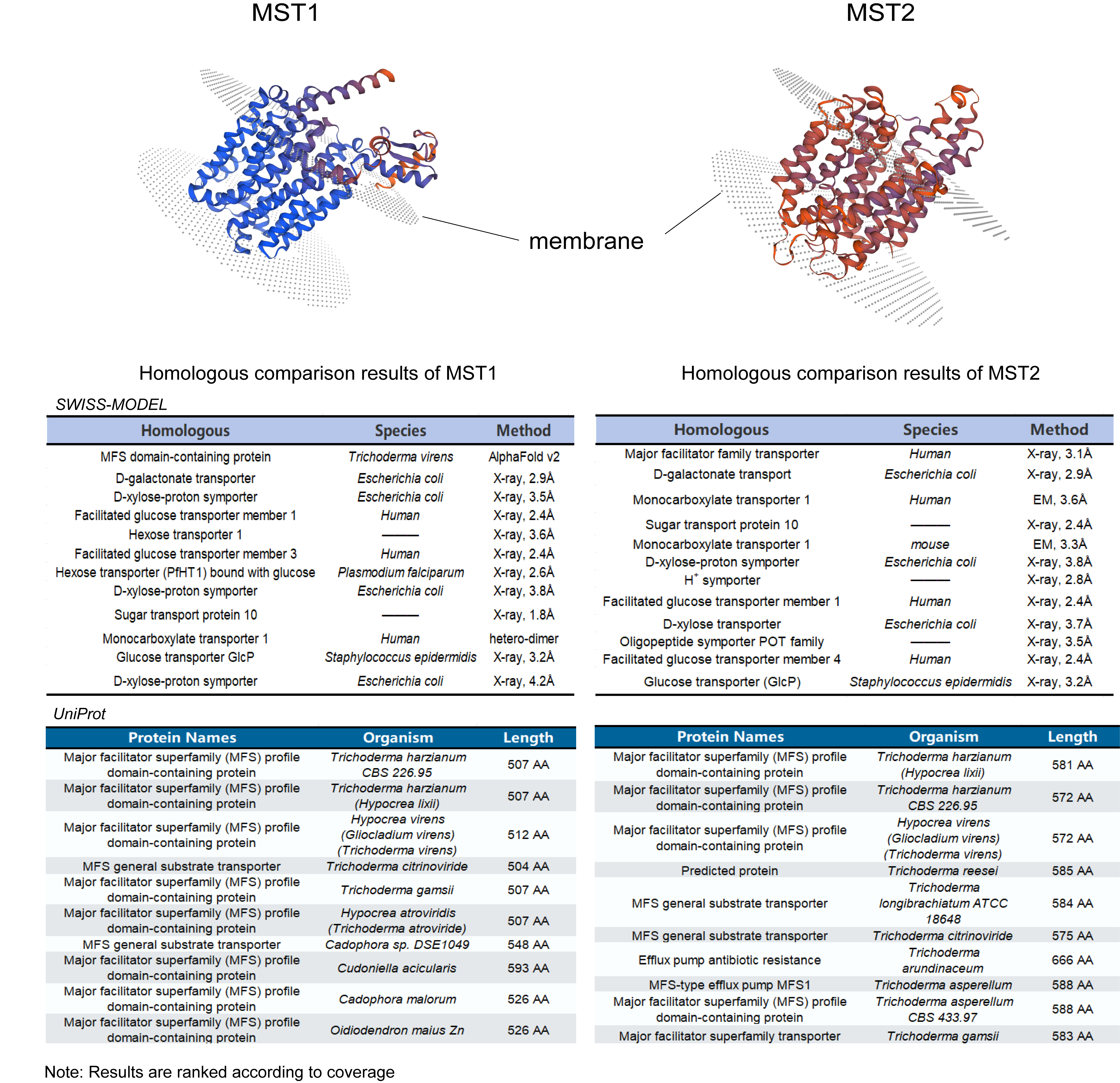


**Fig. S10 Homologous of MST1 and MST2.** The two tables above respectively showed the homologous proteins of MST1 and MST2, by using *SWISS-MODEL*, and the two tables below were the results of the *UniProt* comparison. These results indicated that both MST1 and MST2 had high homology to the glucose transporter, xylose transporter, and hexose transporter.
