# Supplementary material for: Cysteine facilitates the lignocellulolytic response of Trichoderma guizhouense NJAU4742 by indirectly up-regulating membrane sugar transporters
Source: Biotechnol Biofuels Bioprod. 2023 Oct 27;16:159. doi: 10.1186/s13068-023-02418-9 (PMC10612256; doi:10.1186/s13068-023-02418-9)
Supplement: Supplementary file 2 — Additional file 2. Table introduction. [file 13068_2023_2418_MOESM2_ESM.pdf]

Dataset 1A is the differentially expressed gene of OE-*TgcysK* and ∆*TgcysK* relative to WT; **Dataset 1B** is the differentially expressed gene of OE-*Tghmt* and ∆*Tghmt* relative to WT.

Dataset 2A is the Pull-down protein identified in the control (CON) group, while **Dataset 2B** is the Pull-down protein identified in the experiment (EXP) group.

Dataset 3 is the mass spectrometry identification result of intracellular substances of hyphae grown under T1 and T2 treatment conditions.
